# IRS4 induces mammary tumorigenesis and confers resistance to HER2-targeted therapy through constitutive PI3K/AKT-pathway hyperactivation

**DOI:** 10.1038/ncomms13567

**Published:** 2016-11-23

**Authors:** Gerjon J. Ikink, Mandy Boer, Elvira R. M. Bakker, John Hilkens

**Affiliations:** 1 Division of Molecular Genetics, The Netherlands Cancer Institute, Plesmanlaan 121, CX 1066 Amsterdam, The Netherlands

## Abstract

In search of oncogenic drivers and mechanisms affecting therapy resistance in breast cancer, we identified *Irs4*, a poorly studied member of the insulin receptor substrate (IRS) family, as a mammary oncogene by insertional mutagenesis. Whereas normally silent in the postnatal mammary gland, *IRS4* is found to be highly expressed in a subset of breast cancers. We show that *Irs4* expression in mammary epithelial cells induces constitutive PI3K/AKT pathway hyperactivation, insulin/IGF1-independent cell proliferation, anchorage-independent growth and *in vivo* tumorigenesis. The constitutive PI3K/AKT pathway hyperactivation by IRS4 is unique to the IRS family and we identify the lack of a SHP2-binding domain in IRS4 as the molecular basis of this feature. Finally, we show that IRS4 and ERBB2/HER2 synergistically induce tumorigenesis and that *IRS4*-expression confers resistance to HER2-targeted therapy. Taken together, our findings present the cellular and molecular mechanisms of IRS4-induced tumorigenesis and establish IRS4 as an oncogenic driver and biomarker for therapy resistance in breast cancer.

## Abstract (web summary)

IRS proteins are scaffolds that can activate survival signalling pathways. In this study, the authors identified IRS4 as a potential oncogene in breast cancer that leads to the constitutive activation of PI3K/AKT signalling and thus confers resistance to HER2-targeted therapy.

## Graphical abstract

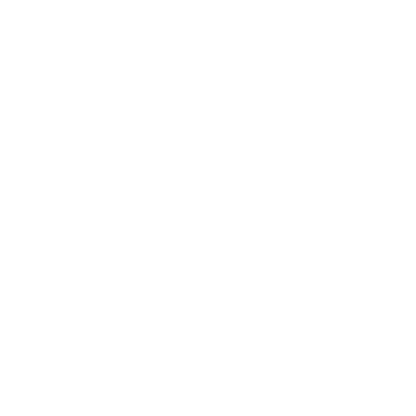

The identification of oncogenes and their associated pathways is crucial for understanding therapy resistance and to develop effective treatment modalities. Insertional mutagenesis is an efficient tool to identify new oncogenes^1^,^2^. We recently discovered several novel proto-oncogenes in high throughput mouse mammary tumour virus (MMTV)-induced insertional mutagenesis screens^3^,^4^, including the insulin receptor substrate 4 (*Irs4*).

The IRS family consists of four closely related members, IRS1–IRS4, and two distant relatives, IRS5/DOK4 and IRS6/DOK5. IRS1 and IRS2 are the best studied members and are shown to have important roles in cell proliferation, survival, migration, metabolism and differentiation, and have been implicated in cancer^5^,^6^,^7^. The *Irs3* gene is only found in rodents and is a pseudogene in humans^8^. *IRS4* was first identified and characterized in the HEK293 human embryonic kidney cell line in which it was shown to undergo rapid tyrosine phosphorylation in response to insulin^9^,^10^.

IRSs are cytoplasmic scaffolding proteins that act as signal transmitters between multiple receptor tyrosine kinases (RTK), including the IGF1 and insulin receptors, and several other Src homology 2 (SH2) domain-containing proteins (reviewed in refs 7, 11). On binding ligand-activated RTKs, numerous tyrosine residues in the large C-terminal region of IRSs are phosphorylated. These phosphorylated tyrosine sites can subsequently serve as binding sites for downstream cytoplasmic SH2-containing effector proteins, including p85 and GRB2, leading to the activation of the PI3K/AKT and MAPK/ERK signalling pathways, respectively (reviewed in refs 5, 11, 12). In addition, it has been reported that phosphorylation of two specific tyrosine residues in the C-terminus of IRS1 and IRS2 leads to binding of tyrosine phosphatase SHP2, providing a negative feedback loop by dephosphorylating the tyrosine residues responsible for, for example, p85 binding^13^,^14^,^15^,^16^,^17^.

In this study, we establish *Irs4* as a novel mammary oncogene and we show that absence of negative feedback regulation in IRS4 leads to constitutive PI3K/AKT-signalling, which functionally differentiates it from IRS1 and IRS2. Next, we demonstrate that IRS4 is expressed in a subset of human breast cancers, collaborates with HER2 to drive tumorigenesis, and confers resistance to HER2-targeted therapy.

## Results

### *Irs4* is targeted by MMTV proviral integration

**Figure 1:**
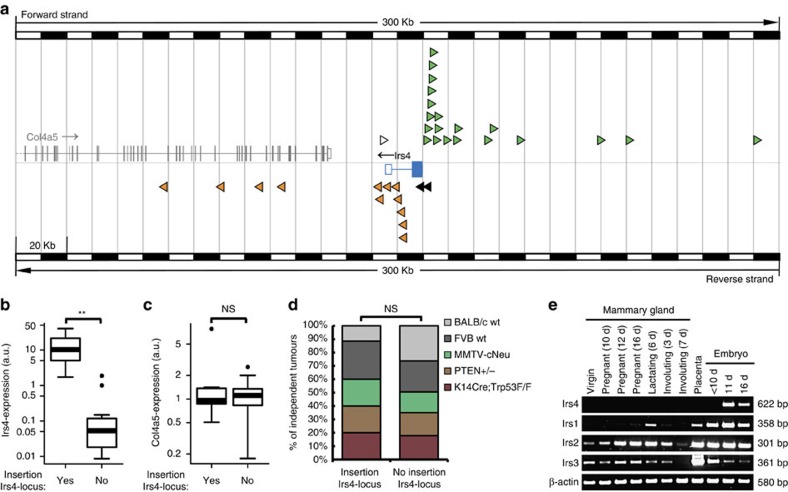
MMTV proviral insertions in the *Irs4* locus and expression analysis. (**a**) Insertion map of a 300 Kb section of the X-chromosome comprising the *Irs4* locus. The map shows the targeted *Irs4* gene (blue) and non-targeted *Col4a5* gene (grey). Rectangles indicate exons, where solid rectangles depict translated exons and open rectangles the UTRs. The lines interspacing the exons indicate introns. Arrowheads indicate the position and orientation (based on Ensembl build 67, NCBIm37) of MMTV proviral insertions in independent tumours, black arrowheads indicate insertions in which the MMTV-LTR putatively will act as a promoter, green or orange arrowheads (upstream or downstream, respectively) indicate insertions potentially acting as enhancer of the endogenous *Irs4*-promoter. One integration in this locus (white triangle) is more likely a spurious insertion improbable to activate *Irs4* (but might activate *Col4a5*). (**b**,**c**) Expression of *Irs4* (**b**) and *Col4a5* (**c**) mRNA in a random series of independent MMTV-induced mammary tumours with (*n*=13) and a random series without (*n*=16) an insertion in the *Irs4* locus, showing a strong correlation between MMTV-insertion in the *Irs4* locus and *Irs4* expression, but no correlation with *Col4a5*-expression. **P<0.01; NS, not significant (Welch's *t*-test). (**d**) The percentage each genotype is contributing to the tumours with (*n*=35) and tumours without (*n*=1,097) insertions in the *Irs4*-locus. No preference for *Irs4* insertions in mammary tumours from any of the tested genotypes: NS, not significant (Pearson's *χ*^2^-test). (**e**) mRNA-expression of *Irs* gene family members at various stages of adult mammary gland development and embryogenesis (d, days) of wildtype BALB/c mice.

We have previously performed high-throughput retroviral insertional mutagenesis screens of MMTV-induced mammary tumours in clinically relevant mouse models of breast cancer and wild-type mice^3^,^4^. From these studies and from a screen performed in mice transgenic for activated rat *ErbB2* (Supplementary Data 1; GJI, MB, ERMB, JH, unpublished data), we obtained MMTV proviral insertion data from a total of 1,132 tumours, of which 35 (3.1%) had insertions that map in the *Irs4*-locus on the X-chromosome. The chromosomal position and orientation of all but one of the insertions is such that either the promoter or enhancer sequences in the proviral LTR can activate *Irs4* (Fig. 1a; Supplementary Data 1). The insertions were unlikely to activate the expression of the adjacent *Col4a5* gene or the 350 kb upstream *Gucy2f*-gene, which is also in opposite orientation to be affected. Indeed, *Irs4* expression, as determined by reverse transcriptase–PCR (RT–PCR) analysis, highly correlated with an MMTV proviral insertion in the *Irs4* locus (*P*=1.61·10^−3^, Welch's *t*-test), while *Col4a5*-expression did not (*P*=0.52, Welch's *t*-test; Fig. 1b,c), which confirms that *Irs4* is indeed an MMTV target and suggests that *Irs4* may act as an oncogene. There was no significant difference of integration frequency in the *Irs4* locus between the tested mouse genotypes (*P*=0.408, Pearson's *χ*^2^-test; Fig. 1d), although this could be due to the low incidence of insertions near *Irs4* in each individual group.

### *Irs4* is the only *Irs* family member targeted by MMTV

While *Irs4* is thus frequently activated by MMTV, we did not find the closely related genes *Irs1*, *Irs2* and *Irs3* as targets in our insertional mutagenesis screens. This suggests that *Irs4* has features that are unique in the *Irs* gene family. Further supporting this, we observed *Irs4* expression restricted to embryonic tissues and adult brain and testis, whereas *Irs1*–*Irs3* were found expressed quite ubiquitous, in accordance with publicly available microarray data (Fig. 1e; Supplementary Fig. 1a,b). In contrast to the other family members, *Irs4* was neither expressed at any stage of post-natal mammary gland development nor in human breast tissue (Fig. 1e; Supplementary Fig. 1c). Also, *IRS4* expression was only observed in two of 25 tested human breast cancer cell lines, MDA-MB-453 and HCC187 cells, and in HEK293 cells (Supplementary Fig. 1d). This limited expression of *IRS4* in human cell lines is in line with publicly available microarray data (Supplementary Fig. 1e). Thus, *Irs4* is a normally silent gene in mammary tissue with potential oncogenic properties unique to its gene family.

### IRS4 acts as oncogenic driver in mammary epithelial cells

**Figure 2:**
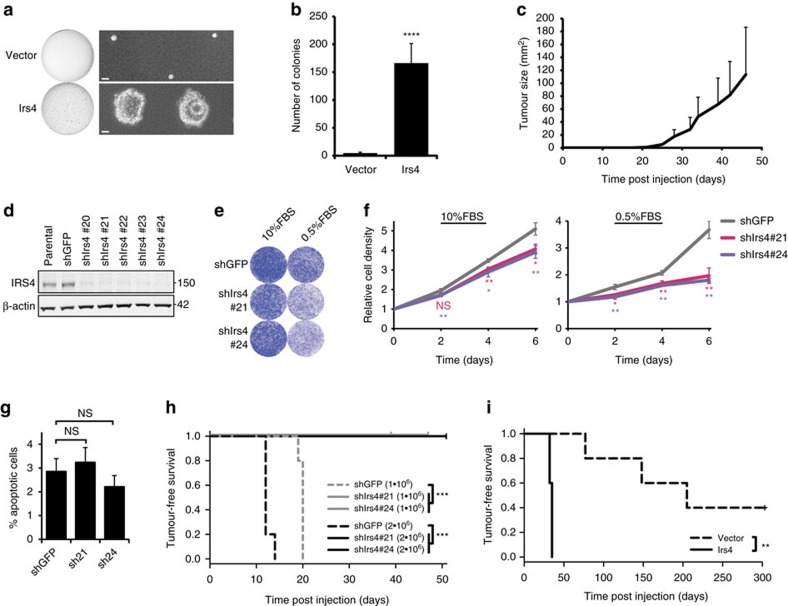
*Irs4* expression in mammary cells drives tumour growth. (**a**) Soft agar growth of MCF10A cells, stably transfected with *Irs4* or empty vector, allowed to grow in soft agar for two weeks. Representative whole well scans and phase-contrast micrographs of three experiments are shown (scale bar, 30 μm). (**b**) Quantification of anchorage-independent growth (mean+s.d.) from three soft agar assays as shown in **a**, using independently transduced MCF10A-cell cultures and each tested in duplicate. ****P<1 × 10^−4^ (Welch's *t*-test, compared with vector). (**c**) Tumour growth in mice subcutaneously injected in both flanks with 1 × 10^6^ P3724-R4 tumour cells (*n*=2, that is, 4 flanks). Data are represented as mean+s.d. (**d**) Western blot of lysates from P3724-R4 cells transduced with the indicated shRNAs showing IRS4-knockdown efficiency at the protein-level. (**e**,**f**) Proliferation rate of P3724-R4 cells on knockdown of *Irs4* using shIrs4#21 or shIrs4#24 versus P3724-R4 control cells transfected with a short hairpin for GFP (shGFP), in 10 or 0.5% serum (FBS), showing representative Crystal Violet staining images at day 6 (**e**) and growth curves depicted as the mean±s.d. of three experiments (**f**). NS, not significant; *P<0.05, **P<0.01 (Welch's *t*-test, compared with shGFP). (**g**) Percentage of apoptotic cells (Annexin V-positive) in three independent viable (propidium iodide-negative) populations of 48 h starved P3724-R4 cells as in **e**, determined by FACS. NS, not significant (Welch's *t*-test). (**h**) Tumour-free survival of groups of five mice each, subcutaneously injected with 1 × 10^6^ or 2 × 10^6^ P3724-R4 cells as in **e**. ***P<0.001 (log-rank test). (**i**) Tumour-free survival of mice subcutaneously injected with 1 × 10^6^ NMuMG-cells ectopically expressing *Irs4* versus vector control cells (*n*=5 for each group, that is, 10 flanks). **P<0.01 (log-rank test).

To validate *Irs4* as a genuine oncogene, we transduced full-length *Irs4* complementary DNA (cDNA) into the human mammary epithelial cell line MCF10A (MCF10A-Irs4) and performed a soft agar colony formation assay. The mere expression of *Irs4* in these cells induced anchorage-independent colony formation, while vector control cells did not (*P*=9.25 × 10^−5^, Welch's *t*-test) (Fig. 2a,b), suggesting *Irs4*-induced tumorigenic potential.

To further substantiate the oncogenic capacity of *Irs4*, we derived a stable epithelial cell line (designated P3724-R4) from an MMTV-induced *K14Cre;Trp53*^*F/F*^ mammary tumour from our insertional mutagenesis screen, with a known proviral integration in the *Irs4* locus and a consequently high *Irs4* expression. After confirming tumorigenicity of this P3724-R4 cell line *in vivo*, we used lentiviral shRNA to knockdown *Irs4* levels in these cells (Fig. 2c,d; Supplementary Fig. 2a,b). The two P3724-R4 cultures with the most effective *Irs4* knockdown grew significantly slower than the green fluorescent protein–short hairpin RNA (GFP–shRNA) negative control, especially under low-serum (0.5%) conditions, while apoptosis rates were not affected (Fig. 2e-g; Supplementary Fig. 2c). When 2 × 10^6^ of these cells were subcutaneously injected into both flanks of five female BALB/c nude mice, none of the mice injected with either of the two *Irs4*-knockdowns developed tumours, whereas we observed tumour growth in both flanks of all five mice injected with 2 × 10^6^ GFP–shRNA control cells after 2 weeks (*P*=1.95 × 10^−4^, log-rank test) and in a subsequent experiment with 1 × 10^6^ injected cells after three weeks (*P*=2.51 × 10^−4^, log-rank test; Fig. 2h). Hence, knockdown of *Irs4* abolished the tumorigenic potential of these mammary tumour cells, showing that the cells are dependent on IRS4 for tumour growth *in vivo*.

Next, we transduced the full-length *Irs4* cDNA into the murine mammary epithelial cell line NMuMG (NMuMG-Irs4). These cells do not express *Brk* (*Ptk6*), a breast cancer associated gene previously shown to interact with *Irs4* (ref. 18). On subcutaneous injection in both flanks of five female BALB/c nude mice, tumour growth was observed in all flanks injected with NMuMG-Irs4 about five weeks post-injection, whereas the mice injected with vector control cells remained tumour-free for more than 10 weeks (*P*=3.13 × 10^−3^, log-rank test), after which tumour growth was sporadically observed in a single flank (Fig. 2i; Supplementary Fig. 2d). Collectively, these results confirm that *Irs4* is an oncogenic driver when expressed in mammary epithelial cells.

### IRS4 causes growth-factor independent cell proliferation

**Figure 3:**
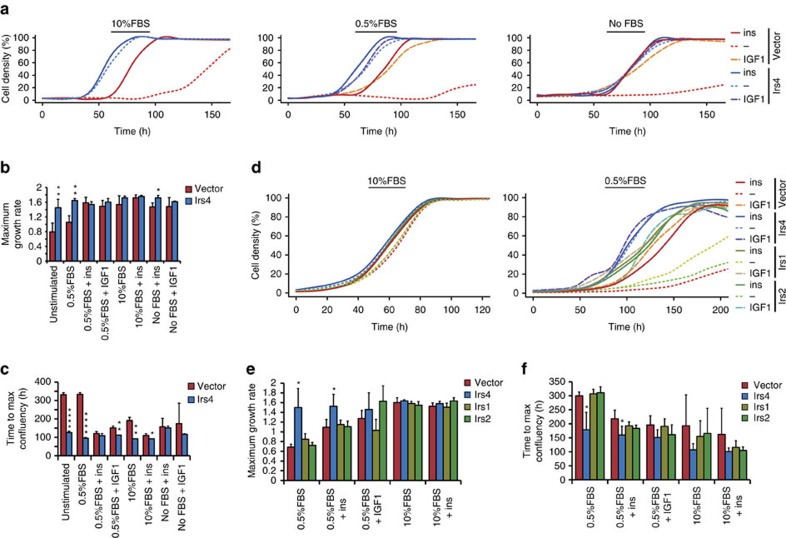
Growth factor-independent cell proliferation in mammary cells expressing *Irs4*. (**a**) Growth curves of MCF10A-cells, stably transfected with *Irs4* or empty vector, growing in the presence of the indicated serum (FBS) concentrations, supplemented with insulin (ins) or IGF1, or without supplement (−). Cell growth is represented as the local regression over quadruplicates. Results shown are representative for four independent experiments, wherein independently transduced cell cultures were used. (**b**,**c**) Quantification of maximum growth rates (**b**) and times until maximum confluency was reached (**c**) derived from growth curves in **a** and determined as outlined in Supplementary Fig. 3a. Data represented as mean+s.d. of quadruplicates. *P<0.05, **P<0.01, ****P<1 × 10^−4^ (Welch's *t*-test, compared with vector in each condition). (**d**) Growth curves of NMuMG-cells, stably transfected with *Irs1, Irs2, Irs4* or empty vector. Conditions and data represented as outlined in **a**. Results shown are representative for two independent experiments. (**e**,**f**) Quantification of maximum growth rates (**e**) and times until maximum confluency was reached (**f**) derived from growth curves in **d**. Data represented as mean+s.d. of quadruplicates. *P<0.05 (Welch's *t*-test, compared with vector in each condition).

Assessing the cellular basis of the oncogenic properties of IRS4, we found that proliferation of the MCF10A-Irs4 cells was higher primarily under conditions without insulin or IGF1, compared with vector control cells (Fig. 3a–c; Supplementary Fig. 3a). The effect on proliferation was most apparent in growth medium containing low (0.5%) or no foetal bovine serum (FBS), as FBS contains these growth factors. In fact, proliferation of cells expressing *Irs4* was hardly affected by the removal of stimuli, in contrast to vector controls which only showed sporadic growth (Supplementary Fig. 3b). Similar observations were made for NMuMG cell proliferation (Supplementary Fig. 3c,d), although these cells required at least 0.5% FBS for growth. This growth factor-independent proliferation capacity appears unique to *Irs4* in the *Irs*-gene family as these mammary epithelial cells endogenously express *Irs1* and *Irs2* (Supplementary Fig. 3e). To exclude the possible effect of expression-level differences, we stably transduced *Irs1* and *Irs2* into NMuMG cells and observed that the cell proliferation rates of these cells were still similar to vector control cells, and when cultured without addition of insulin or IGF-I only very few colonies grew out (Fig. 3d-f; Supplementary Fig. 3f). Thus, the mammary epithelial cells remained insulin/IGF-I dependent even when *Irs1* or *Irs2* were overexpressed, confirming that growth factor-independent cell proliferation is exclusively induced by *Irs4* expression.

### IRS4 induces constitutive PI3K/AKT pathway hyperactivation

**Figure 4:**
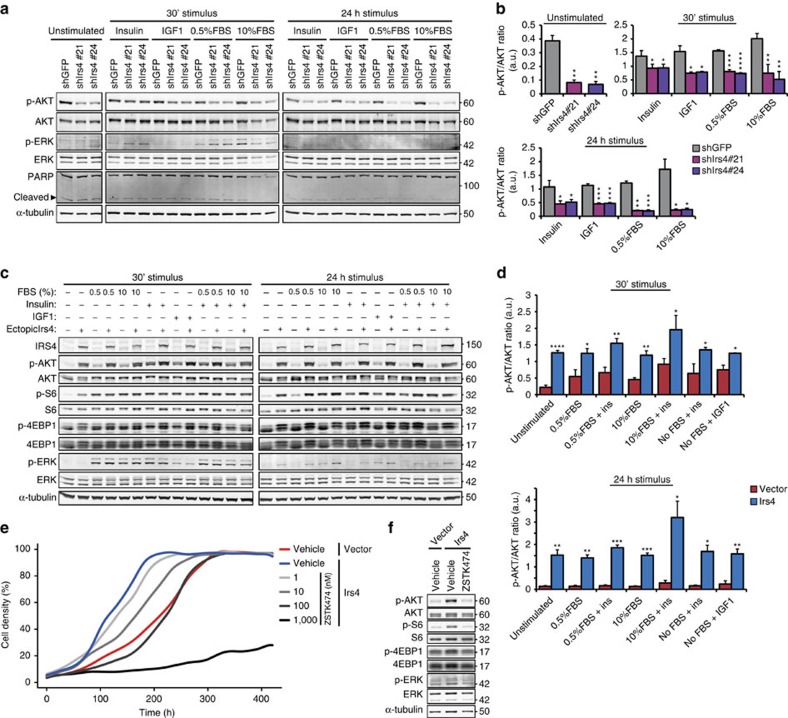
IRS4 constitutively stimulates PI3K/AKT-pathway signalling in mammary cells. (**a**) Western blot showing phosphorylated (p-) and total AKT and ERK, and (cleaved) PARP, from P3724-R4 cells transduced with the indicated shRNAs, under starved (unstimulated) conditions and after stimulation as indicated. (**b**) Ratios of phosphorylated AKT over total AKT (mean+s.d.), quantified from three Western blots as shown in **a**. *P<0.05, **P<0.01, ***P<0.001, ****P<1 × 10^−4^ (Welch's *t*-test, compared with shGFP control in each condition). (**c**) Representative Western blot of three experiments, showing phosphorylated (p-) and total-protein components in the PI3K/AKT and MAPK/ERK-pathways in MCF10A-Irs4 cells (ectopic Irs4: +) or vector controls (-) subjected to the indicated stimuli for 30 min or 24 h. (**d**) Ratios of phosphorylated AKT over total AKT (mean+s.d.) from three blots as shown in **c**. For each blot, we employed independently transduced cell cultures of each cell type shown. *P<0.05, **P<0.01, ***P<0.001, ****P<1 × 10^−4^ (Welch's *t*-test, compared with vector in each condition). (**e**) Growth curves of NMuMG-Irs4 cells in medium supplemented with 0.5%FBS and in the presence of increasing concentrations of the PI3K-specific inhibitor ZSTK474. Growth represented as the local regression over quadruplicates. (**f**) Representative Western blot of two independent experiments, showing phosphorylated and total protein of components in the PI3K/AKT and MAPK/ERK pathways in NMuMG-Irs4 cells cultured 48 h in medium supplemented with 0.5% FBS and 100 nM ZSTK474 or vehicle (DMSO), and vector controls.

Upon knockdown of *Irs4* in the P3724-R4 cells, we observed a robust reduction in AKT-phosphorylation under all tested conditions, while no clear effect was observed on ERK-phosphorylation levels or apoptosis signalling (Fig. 4a,b; Supplementary Fig. 4a). Similarly, we observed a potent growth factor-independent activation of the PI3K/AKT pathway on ectopic *Irs4* expression in MCF10A cells, but no significant effect on MAPK/ERK pathway activation (Fig. 4c,d; Supplementary Fig. 4b,c). Interestingly, MCF10A-Irs4 cells still exhibited high PI3K/AKT pathway activity under all tested conditions after 24 h, whereas the initial FBS and/or insulin/IGF1-induced AKT phosphorylation seen after 30 min in MCF10A vector cells had returned to basal levels at the 24 h time point (Fig. 4d). Similarly, *Irs4* expression in NMuMG cells did not activate the MAPK/ERK pathway, but did stimulate the PI3K/AKT pathway largely independent of any agonist, while NMuMG vector control cells and *Irs1* and *Irs2* transduced cells required stimuli to exhibit any PI3K/AKT pathway activity (Supplementary Fig. 4d–f). The strong and sustained activation of the PI3K/AKT pathway, without requiring extracellular stimuli, implies that IRS4 is a constitutive activator of this pathway, which is likely to account for the IRS4-induced growth factor-independent cell proliferation.

Inhibition of the PI3K/AKT pathway in NMuMG-Irs4 cells using the PI3K-specific inhibitors ZSTK474 (ref. 19) or GDC0941 (ref. 20) reduced cell proliferation in a concentration-dependent manner (Fig. 4e; Supplementary Fig. 5a–d). Specifically, when PI3K/AKT pathway activation was brought down to vector control levels, as determined by AKT phosphorylation, NMuMG-Irs4 exhibited a proliferation rate identical to that of NMuMG vector control cells (Fig. 4f; Supplementary Fig. 5e–k), consistent with the notion that *Irs4* expression regulates cell proliferation through constitutive PI3K/AKT pathway hyperactivation.

### IRS4 lacks negative feedback regulation by SHP2

**Figure 5:**
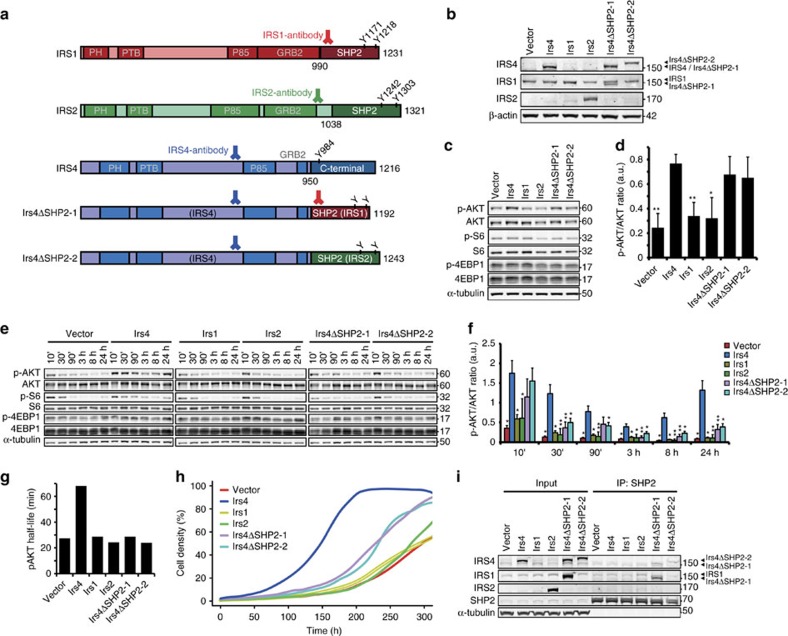
Lack of functional SHP2-binding domain permits constitutive signalling of IRS4. (**a**) Schematic representation of the signalling domains in the IRS-family proteins and recombinant IRS4-proteins containing the SHP2-domains of IRS1 (Irs4ΔSHP2-1) or IRS2 (Irs4ΔSHP2-2), showing the total amino acid length of each product. Locations of tyrosine residues (Y) in the (putative) SHP2-binding motifs and approximate locations of designated antibodies' epitopes are shown. Start positions of the SHP2 domains in IRS1/IRS2 and the C-terminal region in IRS4 that was replaced in the recombinants are indicated. PH, Pleckstrin homology domain; PTB, phosphotyrosine-binding domain. (**b**) Western blot of NMuMG-cells stably transduced with the indicated wildtype *Irs*-family members or *Irs4*-recombinants, and vector controls. IRS4 and IRS1 antibodies, of which the binding sites are depicted in **a**, also bind the differently sized recombinant proteins. (**c**) Representative Western blots of three experiments, showing phosphorylated (p-) and total-protein PI3K/AKT pathway components of the cells as in **b**, after 24 h starvation. (**d**) Ratios phosphorylated AKT over total AKT, calculated from Western blots as shown in **c**. Data are represented as mean+s.d. from three blots using independently transduced cells. *P<0.05, **P<0.01 (Welch's *t*-test, compared with Irs4). (**e**) Representative Western blots of three experiments, showing phosphorylated (p-) and total-protein PI3K/AKT-pathway components in cells as in **b** after stimulation with 0.5%FBS for the indicated time periods. (**f**) Ratios phosphorylated AKT over total AKT, shown as the mean+s.d. from three independent blots as the one shown in **e**. *P<0.05, **P<0.01 (Welch's *t*-test, compared with Irs4 in each time point). (**g**) Estimated half-life of PI3K/AKT pathway signalling (pAKT) after reaching peak AKT phosphorylation levels at 10 min stimulation, determined from three independent blots of **f**, calculated as shown in Supplementary Fig. 6f. (**h**) Growth of the cells described in **b** in 0.5% FBS, represented as the local regression over quadruplicates, representative of two independent experiments. (**i**) Western blot of IRS1, IRS2 and chimeric IRS4 variants co-immunoprecipitated (IP) with SHP2 from lysates (input) of the cells described in **b**. SHP2 and α-tubulin were used as control for IP-specificity and loading control. Results are representative of three experiments using independently transduced NMuMG cells.

From a structural perspective, IRS4 is a unique IRS-protein in its absence of a SHP2-binding motif (Fig. 5a). To investigate whether this could account for the IRS4-specific constitutive PI3K/AKT pathway activation, we replaced the C-terminal region of IRS4 with those of IRS1 or IRS2 containing a SHP2-binding domain (Fig. 5a; Supplementary Fig. 6a). The resulting chimeric proteins, stably expressed in NMuMG cells (NMuMG-Irs4ΔSHP2-1 and NMuMG-Irs4ΔSHP2-2; Fig. 5b; Supplementary Fig. 6b,c), were fully active, as observed by the strong PI3K/AKT pathway activation under unstimulated conditions and after stimulation with 0.5% FBS for 10 min (Fig. 5c,d; Supplementary Fig. 6d,e). Over time, however, phosphorylation of PI3K/AKT pathway components clearly decreased faster in stimulated NMuMG-Irs4ΔSHP2-1 and NMuMG-Irs4ΔSHP2-2 than in NMuMG-Irs4 cells (Fig. 5e,f). In fact, the relative reduction rate and half-life of PI3K/AKT pathway signalling in the IRS4 recombinants was comparable to those in the vector controls and *Irs1*- and *Irs2*-transduced cells (Fig. 5g, Supplementary Fig. 6f). In other words, cells expressing the chimeric IRS4 proteins harbouring the active SHP2 domain, were similarly responsive to negative feedback on AKT phosphorylation as the NMuMG vector, NMuMG-Irs1 and NMuMG-Irs2 cells.

In accordance with the reduction in AKT phosphorylation, we observed a reduced proliferation of the cells expressing the *Irs4* recombinants, compared with cells expressing the wild-type *Irs4* (Fig. 5h; Supplementary Fig. 6g,h). Cell proliferation, however, was not completely reduced to control levels, suggesting that IRS4 also provides some basal constitutive signalling activity additional to the activity sensitive to negative feedback acting on other IRS molecules. Still, replacement of the IRS4 C-terminal domain with the SHP2 domains of IRS1 or IRS2 provides a feedback mechanism that largely quenches IRS4 signalling, most likely due to binding of SHP2, resulting in tyrosine dephosphorylation of the chimera.

To confirm SHP2 binding to the chimeric IRS4, but not to the wild-type IRS4, we performed co-immunoprecipitation assays using a SHP2 antibody and the transduced NMuMG cells. In accordance with previous studies, IRS1 and to a lesser extent also IRS2 co-immunoprecipitated with SHP2, whereas IRS4 did not (Fig. 5i)^13^,^21^,^22^. However, SHP2 was observed to bind the chimeric IRS4 variants and is thus likely to dephosphorylate the IRS4 moiety in these chimeric proteins, similar to what has been reported for IRS1 and IRS2. Collectively, these results provide a mechanism for the lack of negative feedback response of IRS4 signalling and the constitutive activation of the PI3K/AKT pathway in cells expressing *Irs4*.

### *IRS4*-positive breast cancer is associated with poor outcome

**Figure 6:**
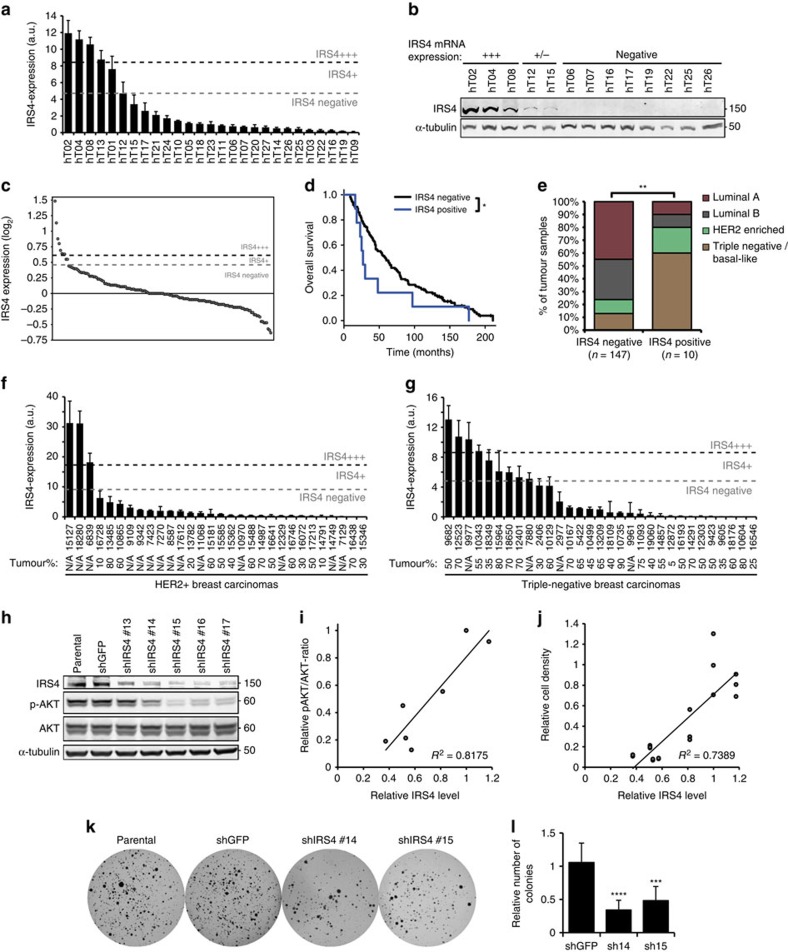
IRS4 in human breast cancer patient material and breast cancer cells. (**a**) Expression of *IRS4*-mRNA in 27 random human primary breast carcinomas (by qRT–PCR). Expression levels greater than median+s.d. (grey dashed line) were defined as positive (IRS4+) and greater than median+2 × s.d. (black dashed line) as highly positive (IRS4+++). (**b**) IRS4 Western blot of a subset of tumours from **a**. +++ are *IRS4*-high samples, +/− are samples at the borderline of *IRS4*-positive and *IRS4*-negative in **a**. (**c**) *IRS4* mRNA*-*expression levels (log_2_ transformed) in 157 metastasized human primary breast carcinomas (microarray probe ILMN_1712774 of Illumina HumanHT-12 v4 Expression BeadChip). Log_2_-ratios greater than 1.5 × s.d. (grey dashed line) were defined as positive (IRS4+) and greater than 2 × s.d. (black dashed line) as highly positive (IRS4+++). (**d**) Overall survival of patients with *IRS4*-negative and *IRS4*-positive (combining IRS4+ and IRS4+++) tumours using the threshold depicted in **c**. **P<0.01 (log-rank test). (**e**) Distribution of the *IRS4*-negative and *IRS4*-positive (combining IRS4+ and IRS4+++) tumours using the threshold depicted in **c** over the indicated PAM50-based clinical subtypes. **P<0.01 (Pearson's *χ*^2^-test). (**f**,**g**) *IRS4*-mRNA expression in 30 HER2-positive (**f**) and 31 triple-negative primary breast carcinomas (**g**), determined by quantitative RT–PCR showing. Expression level cut-offs as in **a**. The tumour percentage (estimated by a pathologist) of each sample is indicated below. N/A indicates that tumour percentage was not available. (**h**) Western blot showing IRS4 protein, and phosphorylated (p-) and total AKT from MDA-MB-453 cells transduced with the indicated shRNAs. (**i**) Quantified IRS4-levels plotted against ratios of phosphorylated AKT over total AKT, derived from the blot in **h**. Linear regression and associated *R*^2^-values show a positive correlation. (**j**) Quantified IRS4 levels, derived from the blot in **h**, plotted against the corresponding cell proliferation rates, determined in triplicate by the Crystal Violet staining as in Supplementary Fig. 7f. Linear regression and associated *R*^2^-values show a strong positive correlation. (**k**) Representative soft agar growth scans of MDA-MB-453 cells, transduced with the indicated shRNAs, cultured for two weeks in absence of insulin, or vehicle. (**l**) Anchorage-independent growth (mean+s.d.), relative to Parental cells, of four independent experiments as in **k**. ***P<0.001, ****P<1 × 10^−4^ (Welch's *t*-test, compared with shGFP).

We examined *IRS4* expression in a random set of 27 human primary breast carcinomas and found five (19%) positive samples, of which four (15%) were highly positive (Fig. 6a). Gene expression levels correlated well with protein levels, though suggested more IRS4-positive samples (Fig. 6b, Supplementary Fig. 7a), and showed a concomitant hyperactivation of the PI3K/AKT pathway (Supplementary Fig. 7b,c). To investigate whether high *IRS4* expression was associated with survival, we examined a well annotated microarray data set obtained from a series of 157 human primary breast tumours from patients that developed metastatic disease^23^. 10/157 (6%) of the tumours showed an elevated *IRS4* microarray signal, which was associated with poor overall survival (*P*=0.0481, log-rank test; Fig. 6c,d). *IRS4*-positive tumours were enriched among triple-negative/basal-like (6/25, 24%) and HER2-enriched tumour subtypes (2/18, 11%), whereas virtually absent in the luminal A (1/67, 1%) and B (1/47, 2%) subtypes (*P*=2.68 × 10^−3^, Pearson's *χ*^2^-test; Fig. 6e; Supplementary Data 2). This subtype enrichment was also evident in the eight (5%) samples that expressed high (+++) *IRS4* levels (*P*=2.13 × 10^−3^, Pearson's *χ*^2^-test; Supplementary Fig. 7d). Moreover, one of the four highly *IRS4*-positive samples in the random set of 27 human primary breast carcinomas was also one of the four *ERBB2*-positive samples (Supplementary Fig. 7e). To expand these data, we screened for *IRS4* expression in an independent uniform HER2-positive and an independent uniform triple-negative human breast cancer series by quantitative RT–PCR (qRT–PCR). From the 30 tested HER2-positive samples, 3 (10%) were highly positive for *IRS4* expression (Fig. 6f). From the 31 tested triple-negative tumours, 9 (29%) could be scored as *IRS4* positive and four (13%) of these as highly positive (Fig. 6g). This is likely an underestimation as the tumour percentage of the samples, as determined by a pathologist, was mostly below 60% (where known), and in some samples as low as 5–10% (Fig. 6f,g).

In line with this clinical data, the two *IRS4*-positive human breast cancer cell lines we identified, MDA-MB-453 and HCC187 (Supplementary Fig. 1d), are both of the triple-negative subtype. Further investigating the involvement of *IRS4* in human breast cancer, we performed a knockdown of the endogenous *IRS4* in these cancer cells and observed a concomitant reduction in PI3K/AKT pathway activation and cell growth (Fig. 6h–j; Supplementary Fig. 7f–i). Moreover, anchorage-independent growth of MDA-MB-453 cells was significantly less in *IRS4* knockdown cells, compared with shGFP-control and parental cells (*P*=9.09 × 10^−5^ and 6.47 × 10^−4^ for shIRS4#14 and shIRS4#15, respectively, Welch's *t*-test; Fig. 6k,l). HCC197 cells did not grow in soft agar at all, in our hands. Collectively, our data demonstrate that *IRS4* is expressed in a subset of human breast tumours and human cancer cell lines. In the light of our finding that IRS4 is constitutively active, these data strongly point to a role for *IRS4* as oncogene in human breast cancer, which may particularly be true in the triple-negative and HER2-positive subtypes.

### IRS4 synergizes with HER2 and confers therapy resistance

**Figure 7:**
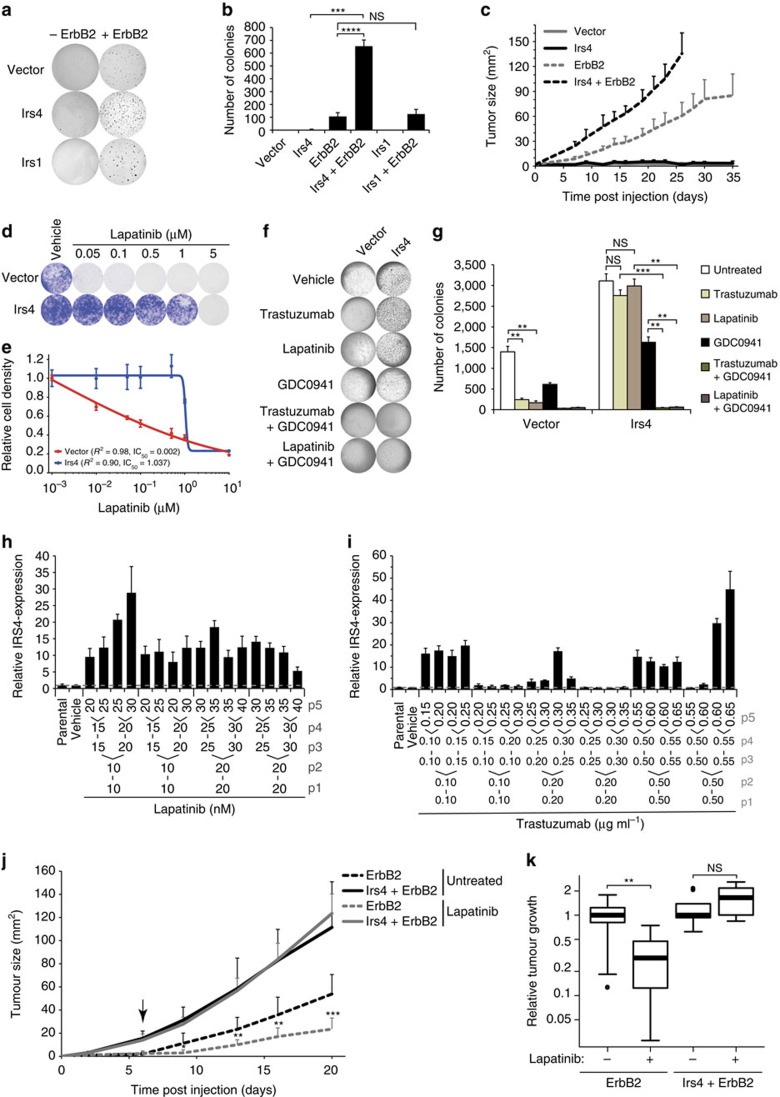
IRS4 synergizes with HER2 and induces resistance to Trastuzumab and Lapatinib. (**a**) Representative soft agar growth scans of NMuMG-cells, stably transduced with *Irs4*, *Irs1* or empty vector only or additionally with activated *ErbB2*, allowed to grow for one week. (**b**) Anchorage-independent growth (mean+s.d.) of three independent experiments as **a**. NS, not significant; ***P<0.001, ****P<1·10^−4^ (Welch's *t*-test, only showing relevant comparisons). (**c**) Tumour growth (mean+s.d.) in mice subcutaneously injected in both flanks with 1 × 10^6^ NMuMG-cells ectopically expressing *Irs4* and/or *ErbB2,* and vector control cells (*n*=5 each, that is, 10 flanks). (**d**) Representative images of four independent experiments, showing Crystal Violet staining of SKBR3-cells, stably transfected with *Irs4* or empty vector, allowed to grow for 11 days in presence of the indicated Lapatinib concentrations, or vehicle. (**e**) Lapatinib dose response curves of SKBR3-cells as in **d**, showing fit over triplicates and correlated *R*^2^-values and IC_50_. Dose response determined as cell proliferation relative to vehicle controls. (**f**) Representative soft agar growth scans of BT474-cells, stably transduced with *Irs4* or empty vector, cultured for 2 weeks in the presence of 5 μg ml^−1^ Trastuzumab or 50 nM Lapatinib with or without 100 nM GDC0941, 100 nM GDC0941 only, or vehicle. (**g**) Quantification of anchorage-independent growth (mean+s.d.) of three independent experiments as in **f**. NS, not significant; **P<0.01, ***P<0.001 (Welch's *t*-test, only showing relevant comparisons). (**h**,**i**) *IRS4* expression levels of BT474 cells (Parental) and those cultured for five passages in presence of increasing Lapatinib (**h**) or Trastuzumab (**i**) concentrations, relative to vehicle-treated cells (grey dashed line). Passage number and drug concentrations are indicated. qRT–PCR data are represented as mean+s.d. of triplicates. (**j**) Tumour growth (mean+s.d.) in mice subcutaneously injected with 1 × 10^6^ *ErbB2*+ NMuMG-cells (as in **c**) in opposite flanks, untreated or treated with 100 mg kg^−1^ day^−1^ Lapatinib (*n*=9 each,that is, 18 flanks). Arrow indicates start of treatment. *P<0.05, **P<0.01, ***P<0.001 (Welch's *t*-test, compared with each untreated control). (**k**) Size of the tumours depicted in **j** at day 20, relative to the corresponding tumour's size at start of treatment. NS, not significant; **P<0.01 (Welch's *t*-test).

To assess the interplay between ERBB2 (HER2) and IRS4, we expressed the activated *ErbB2* oncogene (HER2, neu) in *Irs4*-transduced NMuMG cells and observed a strong synergism in cell proliferation, anchorage independent growth, and *in vitro* tumorigenesis (Fig. 7a–c; Supplementary Fig. 8a). We did not observe this synergism between *ErbB2* and overexpressed *Irs1* (Fig. 7b). Also in the human HER2+ breast tumour cell line BT474, in which *ERBB2* is amplified, *Irs4* expression had a strong effect on anchorage independent growth (*P*=0.0143, Welch's *t*-test; Supplementary Fig. 8b,c).

A major determinant of resistance to HER2-targeted therapy in breast cancer is hyperactivation of the PI3K/AKT pathway^24^,^25^. As we noticed *IRS4* expression particularly in HER2+ tumours and found IRS4 to induce constitutive PI3K/AKT pathway activation, we investigated the involvement of IRS4 with regard to sensitivity to therapeutic agents. Ectopic expression of *Irs4* in human HER2+ breast cancer cell lines SKBR3 and BT474, clearly induced resistance to Trastuzumab and Lapatinib (Fig. 7d,e; Supplementary Fig. 8d–f). To confirm that this resistance is due to PI3K/AKT pathway hyperactivation, we treated the *Irs4*-transduced HER2+ breast cancer cell lines with a combination of Trastuzumab or Lapatinib and the PI3K-inhibitor GDC0941. Indeed, addition of the PI3K inhibitor abrogated *Irs4*-mediated resistance. In fact, combining Trastuzumab or Lapatinib with GDC0941 inhibited cell proliferation rates and anchorage-independent growth much stronger than the single-agent treatments (Fig. 7f,g; Supplementary Fig. 8g,h).

To assess whether IRS4-mediated resistance would occur spontaneously in HER2+ breast cancer cell lines, we cultured naive cell lines for five passages in medium containing increasing concentrations of Trastuzumab or Lapatinib. Expression of *IRS4* was induced within five passages in most of the cultures, especially with Lapatinib, which was not observed when cells were cultured for five passages without the therapeutic agents (Fig. 7h,i; Supplementary Fig. 8i–k). Hence, Trastuzumab and Lapatinib exert a selective pressure in favour of resistant *IRS4*-positive cells. Subsequent knockdown of *IRS4* in the Trastuzumab-resistant cells with the highest endogenous *IRS4*-expression (right-most cultures of Fig. 7i and Supplementary Fig. 8j, designated SKBR3/R^IRS4+^ and BT474/R^IRS4+^, respectively) led to a reduced PI3K/AKT pathway activation and cell proliferation (Supplementary Fig. 9a–d). Knockdown of *IRS4* additionally caused these cell cultures to lose Lapatinib resistance (Supplementary Fig. 9e,f), further supporting the notion that Trastuzumab and Lapatinib resistance can both be IRS4 induced.

To confirm therapy resistance *in vivo*, we subcutaneously injected NMuMG cells, transduced with the activated *ErbB2*-oncogene, in one flank of 18 BALB/c nude mice and NMuMG cells additionally transduced with *Irs4* in each opposite flank. After tumours were established in both flanks, nine mice were randomly assigned to receive Lapatinib treatment. Importantly, we observed a significant reduction in NMuMG-ErbB2 tumour growth (*P*=8.75 × 10^−3^, Welch's *t*-test), whereas tumours from cells additionally expressing *Irs4* were unaffected by Lapatinib treatment (Fig. 7j,k).

Together, these results confirm IRS4-induced Trastuzumab and Lapatinib resistance via hyperactivation of the PI3K/AKT pathway and suggest that *IRS4* expression may also cause HER2-targeting therapy resistant relapse in patients.

## Discussion

In this study, we identify *Irs4* as a gene that is frequently targeted by MMTV proviral insertions and we show that *Irs4* expression confers tumorigenic properties to mammary epithelial cell lines *in vivo*. *Irs4* is also found as common insertion site in Moloney murine leukemia virus (MuLV) insertional mutagenesis screens in various types of lymphomas^26^, stressing the relevance of this gene in oncogenesis. In contrast, *Irs1, Irs2* and *Irs3* are not targeted, neither by MMTV nor by MuLV, and are not found as common insertion site in any insertional mutagenesis screen reported in the Retrovirus and Transposon tagged Cancer Gene Database (RTCGD)^27^. This suggests that IRS4 has unique properties compared with the other IRS family members, especially since all IRS proteins act primarily in the same pathways.

**Figure 8:**
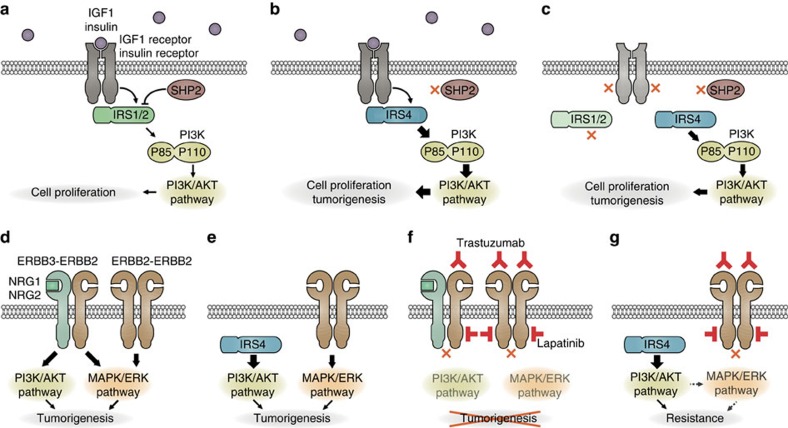
Proposed model of IRS4-induced signalling and therapy resistance. (**a**-**c**) Simplified PI3K/AKT signalling cascade in normal mammary cells expressing *IRS1* and *IRS2*, or mammary cells expressing *IRS4*, in presence (**a**, **b**) or absence (**c**) of insulin/IGF1. (**d**,**e**) Model of signalling in HER2+ (ERBB2) breast cancer cells where ERBB3 (HER3) (**d**) or IRS4 (**e**) activates the PI3K/AKT-pathway. (**f**,**g**) Model of the effect of Trastuzumab or Lapatinib treatment in HER2+ breast cancer cells expressing *ERBB3* (**f**) or *IRS4* (**g**). Thickness of the arrows indicates strength of signalling. Red 'X' indicates no interaction/signalling. Dashed arrow indicates possible cross-talk.

IRS-mediated signalling is initiated upon tyrosine phosphorylation of IRS-proteins by activated RTKs, and leads to activation of the PI3K/AKT and MAPK/ERK pathways (reviewed in refs 11, 28). The importance of IRS signalling in cancer has been reported by Chang *et al*.^29^, showing that IRS1 is constitutively phosphorylated in a variety of human tumours, including breast cancer, and that dominant negative IRS1 abolishes tumour cell growth *in vitro*. However, in light of our findings, IRS1 most likely still requires activation by RTKs, such as the IGF1 receptor, in these tumours. Indeed, high levels of circulating IGF1 have been associated with breast cancer risk^30^ and could well be responsible for activation of IRS1. In contrast, we show that the activity of IRS4 is growth-factor independent, although its activity may be further enhanced by RTKs. Specifically, we demonstrate that IRS4 is an unconventional IRS in that it sustains a high constitutive basal activity in PI3K/AKT-pathway signalling, leading to growth factor-independent cell proliferation in mammary epithelial cells (Fig. 8a-c). Although PI3K/AKT pathway activation by IRS4 has been described before^31^,^32^, the underlying mechanism thus far remained largely unclear.

Phosphorylation of specific tyrosines in the C-terminal domain of IRS1 and IRS2 enables binding of the SH2-containing tyrosine phosphatase SHP2. Despite reported conflicting and context-dependent functions of SHP2 (reviewed in ref. 33), there is ample evidence that SHP2 can bind to IRS1 and IRS2, subsequently induces tyrosine dephosphorylation and thereby reduces signalling activity of these proteins^13^,^14^,^15^,^16^,^17^. IRS4 differs from the other IRS proteins in the absence of a SHP2-binding motif and indeed, SHP2 has been shown not to bind to IRS4 (refs 21, 22), as confirmed by our own experiments. We predicted that the negative feedback loop through tyrosine dephosphorylation of IRS1 and IRS2 by SHP2 may not exist for IRS4 signalling. Indeed, we demonstrate here that an IRS4 protein in which we had replaced the C-terminus with the SHP2-binding domains of IRS1 or IRS2, can no longer maintain the PI3K/AKT pathway hyperactivity as seen with wildtype IRS4 and is even as responsive to negative feedback regulation following stimulation as IRS1 and IRS2. So, unlike IRS1 and IRS2, IRS4-mediated PI3K/AKT signalling is not regulated at the protein level by SHP2, explaining at least in part the constitutive activity of IRS4.

In contrast to the rather ubiquitous expression of *Irs1* and *Irs2*, expression of *Irs4* is very stringently regulated. Restricted expression of a constitutive active gene involved in cell proliferation would be expected, as such gene would render cells more prone to cancer. In fact, the inference that IRS4 is a constitutive active IRS is also in line with our finding that the *Irs4* gene is a target for retroviral insertional mutagenesis, as this process leads with few exceptions to transcriptional deregulation of the target genes rather than to mutations affecting protein function^34^. The notion that IRS4 is a constitutive active oncogenic protein primarily regulated transcriptionally also explains the recent observations that chromosomal translocation events can activate *Irs4* expression, leading to T-cell acute lymphoblastic leukaemia^35^,^36^ and subungual exostosis (a benign bone and cartilage producing tumour)^37^. In addition, in human hepatocellular carcinomas (HCC), *IRS4* expression is frequently upregulated compared with hepatocytes^38^, and its role in this malignancy has been further substantiated in the HEPG2 hepatoblastoma cell line where *IRS4* plays an important role in cell proliferation^39^. These findings suggest that *IRS4* expression is highly relevant in various human cancers.

Our data establish *Irs4* as a potent oncogene in mouse mammary tumorigenesis and we show that *IRS4* expression in human primary breast cancers is associated with poor survival. Furthermore, we observed *IRS4* expression almost exclusively in the triple-negative and HER2+ subtypes, while only incidentally in the Luminal A and B subtypes. Triple-negative tumours are independent of the most common growth stimulatory signals in breast cancer, such as steroid hormones, and require other factors to activate the essential pathways in mammary carcinogenesis, like the PI3K/AKT pathway. Indeed, several mechanisms for PI3K/AKT pathway activation, like *PIK3CA* mutations or copy number gain, AKT activation and *PTEN* loss, have been associated with triple-negative (basal-like) tumours^40^,^41^,^42^,^43^. In HER2+ tumours, the MAPK/ERK pathway is activated through ERBB2, but PI3K/AKT pathway activation is additionally required, for example, via *ERBB3* (*HER3*) upregulation (Fig. 8d), *PIK3CA* mutations or *PTEN* loss^44^,^45^,^46^,^47^,^48^,^49^. In both triple-negative and HER2+ breast cancers, *IRS4* expression is most likely an additional mechanism to activate the PI3K/AKT pathway and consequently, tumorigenesis. Indeed, we found that co-expression of *Irs4* and *ErbB2* in mammary epithelial cells synergistically accelerated tumour growth due to their activity in complementary oncogenic pathways: the PI3K/AKT and MAPK/ERK pathways, respectively (Fig. 8e). Besides the identified oncogenic activities that IRS4 executes via PI3K/AKT pathway hyperactivation, IRS4 might also contribute to oncogenesis via additional mechanisms. The IRS family is known to interact with many proteins, apart from the canonical p85 and GRB2, and can even form large multiprotein complexes (reviewed in refs 7, 50). Moreover, nuclear translocation with associated effects on, for example, DNA repair, rRNA synthesis and gene expression, has been reported for IRS proteins as well^6^,^31^,^51^,^52^,^53^, processes which also play a role in oncogenesis. Therefore, it may be useful to investigate these aspects in IRS4-induced tumours in the future.

Hyperactivation of the PI3K/AKT pathway is frequently found to cause resistance to the ERBB2-targeting monoclonal antibody Trastuzumab (Herceptin) or the small molecule ERBB2 kinase inhibitor Lapatinib (Tykerb) in HER2+ breast cancer^24^,^25^,^54^. This urged us to investigate whether IRS4-mediated constitutive PI3K/AKT pathway activation causes resistance to HER2-targeting drugs in HER2+ breast cancer cell lines (Fig. 8f,g). Our results clearly demonstrate that *IRS4* expression induces resistance to both Trastuzumab and Lapatinib *in vitro* and Lapatinib resistance *in vivo*. In a clinically relevant setting, we moreover demonstrate that treatment of breast cancer cells with suboptimal doses of these HER2-targeting drugs consistently results in selection of cells with upregulated *IRS4*-levels, in particular with Lapatinib. This suggests that *IRS4*-induction, leading to drug resistance, may also frequently occur in patients treated with these anti-cancer drugs.

One may consider treating HER2+ tumours expressing high levels of *IRS4* with drugs targeting PI3K, AKT and/or mTOR in combination with HER2-targeted therapy. However, in the recently completed BOLERO-3 randomized phase-III study of Trastuzumab-resistant advanced breast cancer, the addition of the mTOR-inhibitor Everolimus to Trastuzumab and Vinorelbine treatment showed some clinical benefit only in hormone receptor negative (HR−) patients^55^. The BOLERO-1 phase-III randomized study similarly showed a prolongation of progression-free survival by combined treatment of Trastuzumab with Paclitaxel and Everolimus in patients with HER2+ HR− advanced breast cancer, although this did not reach protocol-specified significance^56^. However, the fact that both these trials did not preselect patients with tumours exhibiting high PI3K/AKT pathway activation may have limited the overall outcome. Indeed, patients from the BOLERO-3 trail with PTEN-low and pS6-high tumours both derived significantly more benefit from Everolimus than the PTEN-high and pS6-low groups, respectively^55^. Hence, clinical trials with selective enrolment are needed to shed more light on the clinical benefits of combined HER2 and PI3K/AKT/mTOR targeted therapy in tumours with PI3K/AKT pathway hyperactivation, including IRS4+ tumours.

Altogether, we propose *IRS4* as a novel clinical biomarker for HER2-targeted therapy resistance. Moreover, *IRS4* may also prove to be of wider clinical relevance as biomarker for PI3K/AKT pathway-dependent breast and other cancers.

## Methods

### Patient samples

We obtained a set of 27 random human primary breast carcinoma samples, without clinical data, from our in-house pathology department. In addition, RNA samples were obtained from 31 tumours randomly selected from a collection of triple-negative primary breast carcinomas^57^, and from 30 tumours randomly selected from a set of 129 HER2-positive primary breast carcinomas. The latter specimens were obtained from patients treated between 1989 and 2006 at The Netherlands Cancer Institute. Microarray data of 157 primary breast tumours that had metastasized^23^ were kindly shared by the group of M.J. van de Vijver (AMC, Amsterdam) and were obtained using the Illumina HumanHT-12 v4 Expression BeadChip, followed by robust spline normalization and ComBAT batch correction. Use of patient material was approved by the individual patients (via opt-out) and by the local Translational Research Board, following positive recommendation of the Medical Ethical Committee.

### Animal experiments

Methods used for the MMTV-induced insertional mutagenesis screens have been described previously^3^,^4^. Mouse models used for MMTV-induced insertional mutagenesis were the FVB and BALB/c wild-type mouse strains and three clinically relevant mouse models of breast cancer: *MMTV-cNeu* (FVB background), transgenic for activated rat ErbB2 (ref. 58); *K14Cre;Trp53*^*F/F*^ (BALB/c background), a p53 conditional knockout^59^; and *PTEN*^*+/*−^ (FVB background), a PTEN heterozygous mouse model^60^.

Tumorigenicity experiments were performed by subcutaneously injecting 1 × 10^6^ or 2 × 10^6^ viable cells in 200 μl PBS into each flank of three to 4 weeks old female *BALB/cABomA-nu/nu* (BALB/c nude) mice. Viability of cells was assessed by Trypan Blue staining and differed neither between cells from the test group and controls, nor before and after injection. Tumour growth was measured at least twice a week in two dimensions and the animals were sacrificed when the tumour reached ∼1 cm^3^. *In vivo* Lapatinib resistance was assessed by subcutaneously injecting 1 × 10^6^ NMuMG-ErbB2 in one flank and 1 × 10^6^ NMuMG-ErbB2-Irs4 cells in the opposite flank, followed by random assignment of the mice to receive normal chow or chow containing Lapatinib, 4 days after observing tumours in both flanks. Lapatinib obtained from our in-house pharmacy was mixed with standard mouse chow (0.48 g kg^−1^) as described before^61^, translating to a dose equivalent to 100 mg kg^−1^ per day. Kaplan–Meier plots of tumour-free survival were plotted using the programming language R with the ‘survival' package. All mouse experiments were performed in accordance with the Dutch legislation and were approved by the Animal Experiments Committee (DEC).

### Insertion site mapping

To map the MMTV insertion sites we used the protocols as previously described^3^,^4^.

### Cell culture and treatment

The *Irs4*-positive tumour cell line P3724-R4 was established from an MMTV-induced mammary tumour that developed in a *K14Cre;Trp53*^*F/F*^ mouse and highly overexpressed *Irs4* due to an MMTV proviral insertion in the *Irs4* locus. 1–2 mm pieces of the tumour were resuspended in a small volume of PBS and subcutaneously injected in BALB/c nude mice (100 μl per flank). Tumours that developed were isolated when reaching about 1 cm^3^ in size, cut into 1–2 mm pieces and incubated for 50 min in 0.25% Collagenase III (Worthington) and 0.2% Hyaluronidase (Sigma-Aldrich) in Dulbecco's modified Eagle's medium (DMEM) (Invitrogen) at 37 °C. Subsequently, the cells were filtered through a 70 μm cell strainer (BD Falcon), centrifuged at 250 g for 5 min, resuspended and cultured in DMEM supplemented with 10% FBS (Perbio), 50 units ml^−1^ penicillin and 50 μg ml^−1^ streptomycin (PenStrep) (Gibco), 200 ng ml^−1^ hydrocortisone (Gibco) and 20 ng ml^−1^ human recombinant epidermal growth factor (EGF) (Sigma-Aldrich), on 2 μg ml^−1^ collagen-I coated Petri dishes. Medium was not supplemented with insulin to select for *Irs4*-positive cells. After selecting for epithelial cells by multiple rounds of differential trypsinization, the immortalized epithelial cells were maintained on 10% FBS coated plastic-ware. Clones of the immortalized cell culture with a clear stable epithelial morphology were isolated and expanded. One of these clones was used for further experiments and was designated P3724-R4, which was further maintained on 10% FBS-coated Petri dishes in DMEM/F-12 mix (Invitrogen) supplemented with 10% FBS, PenStrep, 200 ng ml^−1^ hydrocortisone and 20 ng ml^−1^ EGF. On knockdown of *Irs4*, cells were maintained in medium additionally supplemented with 5 μg ml^−1^ insulin (SAFC Biosciences).

NMuMG, MCF10A, BT474, MDA-MB-453 and HCC1187 cells were obtained from ATCC. SKBR3-cells were a kind gift from J. Taylor-Pappadimitriou (Guy's Hospital, London). All cell lines were never cultured for more than eight passages on receipt and were routinely tested for Mycoplasma (Hoechst staining and PCR).

NMuMG and MCF10A-cells were cultured in DMEM/F-12 (Invitrogen) supplemented with FBS (Perbio), 50 units ml^−1^ penicillin and 50 μg ml^−1^ streptomycin (PenStrep) (Gibco), and 5 μg ml^−1^ insulin (SAFC Biosciences). MCF10A-medium was additionally supplemented with 200  ng ml^−1^ hydrocortisone (Gibco) and 20 ng ml^−1^ human recombinant epidermal growth factor (EGF) (Sigma-Aldrich). BT474 and SKBR3-cells were cultured in DMEM (Invitrogen) supplemented with 10% FBS and PenStrep. SKBR3 medium was additionally supplemented with 5 μg ml^−1^ insulin. MDA-MB-453 and HCC1187 cells were cultured in RPMI 1640 medium (Gibco) supplemented with 10% FBS and PenStrep.

Where indicated, 5 μg ml^−1^ insulin or 100 ng ml^−1^ recombinant human IGF1 (Peprotech) was supplemented to the medium (as stimulus). PI3K-specific inhibitors GDC0941/Pictilisib (Selleck Chemicals) and ZSTK474 (Selleck Chemicals) dissolved in dimethyl sulfoxide (DMSO) were added to the culture medium at the indicated concentrations. Trastuzumab/Herceptin (Roche) dissolved in water was obtained via our outpatient clinic. Lapatinib/Tykerb was purchased from Selleck Chemicals as Lapatinib Ditosylate (GW-572016) and was dissolved in DMSO. Equal volumes of DMSO diluent were added to the medium, where mentioned, as vehicle controls.

All experiments were performed with at least two independently transduced cell cultures each.

### Establishing resistant cell cultures

50,000 BT474 or SKBR3-cells per well were seeded in a 6-wells plate in medium containing 0.1, 0.2 or 0.5 μg ml^−1^ Trastuzumab; 10, 20 or 40 nM Lapatinib; or equal volumes of DMSO diluent. Cells were passaged on reaching near-confluency and half of the cells were transferred to a new well, while the other half was used for RNA isolation. Every second passage, cells were split and transferred into two new wells instead of one. Trastuzumab and Lapatinib concentrations were increased in the next passage if cell proliferation rates were comparable to the vehicle-treated cells, or kept the same when growth was noticeably slower (see Fig. 7h,i; Supplementary Fig. 8i,j for a schematic representation of the concentrations used).

### Plasmids and gene transduction

Five individual shRNAs that target murine *Irs4* and a shRNA targeting GFP cloned in the lentiviral vector pLKO.1 were obtained from the RNAi Consortium (TRC) (Irs4 shRNA clone IDs: TRCN0000105820, TRCN0000105821, TRCN0000105822, TRCN0000105823, TRCN0000105824 and eGFP clone ID: TRCN0000072185, here referred to as shIrs4#20, shIrs4#21, shIrs4#22, shIrs4#23 shIrs4#24 and shGFP, respectively). Knockdown of human *IRS4* was achieved using TRC shRNAs with IDs: TRCN0000063613, TRCN0000063614, TRCN0000063615, TRCN0000063616 and TRCN0000063617, here designated shIRS4#13, shIRS4#14, shIRS4#15, shIRS4#17 and shIRS4#17, respectively. Lentiviral supernatants were produced following transfection as described by the TRC (http://www.broadinstitute.org/rnai/public/resources/protocols). Briefly, 48 h post-transfection, the culture medium of lentivirus 293T packaging cells (lentiviral supernatants) transfected with short hairpin constructs was passed through a 0.45 μm filter (Whatman), supplemented with 6 μg ml^−1^ polybrene (Millipore). shIrs4#20–24 and shGFP were used for transduction into our newly established P3724-R4 tumour cell line and shIRS4#13–17 and shGFP for transduction in SKBR3/R^IRS4+^, BT474/R^IRS4+^, MDA-MB-453 and HCC1187 cells. Cells expressing a shRNA were selected with 2 μg ml^−1^ puromycin, 48 h post infection. shRNA-induced knockdown efficiency was determined by quantitative RT–PCR and immunoblotting after two weeks of puromycin selection.

The pMSCV-construct with wild-type mouse *Irs1* was a kind gift of Dr R. Baserga^62^. The pBABE-construct with wildtype mouse *Irs2* was obtained through Addgene (plasmid 11371) and was originally cloned in the laboratory of Dr R. Kahn^32^. A construct carrying mutant rat *ErbB2/Neu* cDNA was a kind gift of Dr W.J. Muller (McGill Cancer Center, Montreal) and was cloned in a pMSCV vector with blasticidin resistance gene. Mouse wildtype *Irs4* cDNA cloned into a pMSCV vector was kindly provided by Dr A. Berns (NKI, Amsterdam). The *Irs1* and *Irs2*-constructs were used as donors of the SHP2-domains to generate the recombinant Irs4ΔSHP2-1 and Irs4ΔSHP2-2 constructs in pMSCV. The empty pMSCV backbone vector was used as control in each experiment. Ecotropic retroviruses were produced in Phoenix packaging cells, transfected with the appropriate ecotropic retroviral construct using the calcium-phosphate precipitation method. Cells were infected with the ecotropic virus as described above for lentiviral transduction. Infected cell populations expressing the introduced transgene were selected in medium containing 2 μg ml^−1^ puromycin or 5 μg ml^−1^ blasticidin 48 h after infection. Ecotropic retroviral infection of human MCF10A-cells was facilitated by using an oligoclonal MCF10A-cell pool stably expressing the murine ecotropic receptor.

All used retroviral plasmids carried the puromycin resistance gene, with the exception of pMSCV-ErbB2, which carried the blasticidin resistance gene. The integrity of the inserts in each construct was verified by sequencing and expression of the transduced constructs was confirmed by RT–PCR and western blot analysis, following puromycin or blasticidin selection.

### Expression analysis

RNA isolation, RT–PCR and expression analysis of tissues and tumour material was performed as described before^4^. From cell lines, RNA was isolated using the HighPure RNA isolation kit (Roche), followed by DNAse treatment and RT–PCR using the Tetro cDNA synthesis kit (Bioline), following manufacturers' instructions. qPCR was performed using the SensiFast SYBR Hi-ROX kit (Bioline) on a StepOnePlus Real-Time PCR system (Applied Biosystems) and expression levels were determined using the ΔΔCT-method.

MMTV-induced tumours from the insertional mutagenesis screens were assessed by qRT–PCR for *Irs4*-expression (forward primer: 5′-TCCTGTACCAATGCTTCTCCG-3′ and reverse primer: 5′-CGCGAAGTATTCGTCCTGGG-3′) and *Col4a5*-expression (forward primer: 5′-GTCCACCAGGTACAGAAGGTC-3′ and reverse primer: 5′-CTCCTTTCAAACCAGGTAAGCC-3′). β-actin-expression (forward primer: 5′-GGCTGTATTCCCCTCCATCG-3′ and reverse primer: 5′-CCAGTTGGTAACAATGCCATGT-3′) was used as a reference. Quantitative real-time PCR to assess knockdown efficiency of *Irs4* was performed using the same primers for murine *Irs4* and β-actin.

Expression of *Irs1-4* in BALB/c wild-type tissues was assessed by RT–PCR using the following primers: for *Irs1*, forward primer: 5′-TCTCCAAGGAGTCGGCTCCA-3′ and reverse primer: 5′-CGTGAGGTCCTGGTTGTGAA-3′; for *Irs2*, forward primer: 5′-TGGGTTTCCAGAACGGCCTC-3′ and reverse primer: 5′-TTTCAACATGGCGGCGATGG-3′; for *Irs3*, forward primer: 5′-GTACCGTTAGCCTGGAGGGT-3′ and reverse primer: 5′-CTTCCAGGCTTTCGCAGGAG-3′, and for *Irs4*, forward primer: 5′-ATTGCTGCTCCAGCTGAGGC-3′ and reverse primer: 5′-AATGGATGCAGGAGGCAGTC-3′.

Expression analysis in patient samples and human breast cancer cell lines was performed with quantitative RT–PCR using human-specific *IRS4* primers (forward primer: 5′-CGACCAAGCGACAAGAAGACT-3′ and reverse primer: 5′-GGTTCCCGAGGAAAGAAGCG-3′) and human-specific *ERBB2* primers (forward primer: 5′-TGGCCTGTGCCCACTATAAG-3′ and reverse primer: 5′-AGGAGAGGTCAGGTTTCACAC-3′), using expression of β-actin (forward primer: 5′-CCAACCGCGAGAAGATGA-3′ and reverse primer: 5′-CCAGAGGCGTACAGGGATAG-3′) as a reference Although our RNA-isolation procedure included an integrated DNA-digestion step to avoid genomic DNA amplification, primer pairs were designed to span an intron, whenever possible.

All expression analysis results were independently confirmed by at least one alternative primer pair. Product size of PCR reactions (always 30 cycles) is indicated next to each gel. Full images of gels are provided in Supplementary Fig. 10.

### Cell proliferation assays and dose response

For dynamic cell proliferation analysis, 1,500 (MCF10A) or 3,000 (NMuMG) cells per well were allowed to attach overnight in a 10% FBS-coated clear-bottom black 384-well plate (BD Falcon) in DMEM/F-12 medium supplemented with PenStrep, and for MCF10A-cells additionally supplemented with 200 ng ml^−1^ hydrocortisone and 20 ng ml^−1^ EGF. After replacing the medium with the appropriate growth medium, every well was imaged (phase-contrast) with a 4 h interval using the IncuCyte life cell imaging device (Essen BioScience). The changes in cell density over time were used by the IncuCyte software to determine the growth curves. Local regression of confluency over time, and calculations for maximum growth rates and times to reach maximum confluency were performed using the programming language R with the ‘cellGrowth' package. All these experiments were carried out at least in quadruplicate and were independently repeated.

For end-point cell proliferation assays, 30,000 P3724-R4, 25,000 NMuMG, 12,500 SKBR3, 25,000 BT474, 30,000 MDA-MB-453, 30,000 HCC1187, 30,000 SKBR3/R^IRS4+^ or 50,000 BT474/R^IRS4+^ cells per well were seeded in 24-well plates (Corning) for the Trastuzumab and Lapatinib resistance experiments. Cells were allowed to attach for 6 h, washed with DMEM/F-12 medium only supplemented with PenStrep and then grown under the indicated conditions. At indicated time points, the cells were fixed in 4% buffered Formaldehyde solution (Klinipath), stained with 0.1% Crystal Violet solution (Sigma-Aldrich) and imaged using a desktop scanner (Epson). The experiments with SKBR3 or BT474-cells were all performed four times, using independent stably transduced oligoclonal cultures of the cells. The experiments with P3724-R4, SKBR3/R^IRS4+^ and BT474/R^IRS4+^ cells were performed in triplicate.

To assess dose effect of Lapatinib, we monitored cell proliferation of 1,000 SKBR3 cells per well (in quadruplicate) in a clear-bottom black 384-well plate (BD Falcon) subjected to 0.001, 0.01, 0.05, 0.1, 0.5, 1 or 10 μM Lapatinib, or vehicle (DMSO), using the IncuCyte life cell imaging device (Essen BioScience). Dose response curves were fitted by nonlinear regression of cell densities (normalized to vehicle-treated cells) over log-transformed Lapatinib concentration values using the GraphPad PRISM 6 software.

### Apoptosis assays

1.0 × 10^6^ P3724-R4 cells were allowed to attach overnight to a T25 flask (BD Falcon) in DMEM/F-12 medium supplemented with 10% FBS, PenStrep, 5 μg ml^−1^ insulin, 200 ng ml^−1^ hydrocortisone and 20 ng ml^−1^ EGF and were then washed and cultured for 48 h on DMEM/F-12 medium only supplemented with PenStrep (starved). Cells were released by EDTA-free trypsin-250 (Gibco) diluted in DMEM/F-12 medium and immediately stained with Annexin V and propidium iodide (PI) from the Annexin V-FITC Apoptosis Detection Kit (Abcam), following manufacturers' instructions. 20,000 PI-negative cells per replicate were analysed for Annexin V-staining using a Beckton Dickinson LSRII FACS analyser.

### Soft agar assays

Soft agar assays were performed in six-well plates. Each well contained a 2 ml 0.6% low-gelling temperature agarose (Sigma-Aldrich) base layer on which 50,000 MCF10A, 10,000 NMuMG, 10,000 BT474 or 50,000 MDA-MB-453 cells were suspended in 2.5 ml of 0.35% low-gelling temperature agarose in medium (supplemented with Trastuzumab, Lapatinib, GDC0941 or vehicle, where applicable). Anchorage-independent growth was assessed by counting colonies after two weeks using the GelCount instrument (Oxford Optronix). All soft agar assays were performed in at least three separate experiments, using independently transduced cell cultures.

### Immunoprecipitation

Immunoprecipitation of SHP2 from cells lysed in NP40 lysis buffer (50 mM Tris-HCl pH 7.4, 150 mM NaCl, 20 mM EDTA, 1% NP40, Complete Mini protease inhibitor and PhosStop phosphatase inhibitor; both from Roche) was carried out overnight at 4 °C using an anti-SHP2 antibody conjugated to agarose beads from Santa Cruz Biotechnology (#sc-280 AC), following manufacturer's instructions. The immunoprecipitated proteins were solubilized in sample buffer and analysed by 4–12% Bis-Tris gel electrophoresis followed by western blotting.

### Western blotting

Cells were washed twice with PBS and then lysed in NP40 lysis buffer for 30 min on ice. Harvested lysates were then centrifuged at 15,000*g* for 10 min at 4 °C and the protein concentration of the supernatants was determined by the Micro BCA protein assay kit (Thermo Scientific). Equal amounts of protein were separated on 4–12% Bis-Tris gels using the Novex NuPAGE or Bolt electrophoresis systems (Life Technologies) and subsequently transferred onto nitrocellulose membranes (Whatman). Membranes were blocked with Odyssey PBS Blocking Buffer (LI-COR) and immunostained with antibodies against the proteins of interest. Proteins were detected in either the 700 or 800 nm channel using the Odyssey Infrared Imaging System (LI-COR), after incubation with the appropriate secondary antibodies labelled with IRDye 680 or IRDye 800 fluorescent dyes (LI-COR), respectively. Results were quantified using the Image Studio software (LI-COR), where the absolute signals or ratios phosphorylated over total protein signal were always normalized over the loading control signals in each lane. In every Western blot analysis, all proteins analysed in the same experiment were detected on the same blot. Blots were always first immunostained for IRS4, IRS1, p-AKT, p-S6, p-4EBP1, p-ERK, total ERK, SHP2 and/or loading controls (α-tubulin or β-actin), followed by stripping using the Newblot Nitro Stripping Buffer (LI-COR) as recommended by the manufacturer, and subsequently immunostained for IRS2, total AKT, total S6 and/or total 4EBP1.

Antibodies against p-AKT S473 (#4060, 1:2,000), p-MAPK (p-ERK) T202/Y204 (#9106, 1:2000), p-S6 S235/236 (#4858, 1:2,000), p-4EBP1 T37/46 (#2855, 1:1,000), total S6 (#2217, 1:1,000), total 4EBP1 (#9644, 1:1,000), total MAPK (ERK) (#9102, 1:1,000) and PARP (#9542) were obtained from Cell Signalling Technology. Goat anti-IRS4 antibody (#EB11828, 1:4,000) was obtained from Everest Biotech. Antibodies against IRS1 (#sc-559, 1:500), IRS2 (#sc-8299, 1:500), SHP2 (#sc-280, 1:1,000) and total AKT (#sc-8312, 1:1,000) were purchased from Santa Cruz Biotechnology, α-Tubulin (#T9026, 1:5,000) from Sigma-Aldrich, and β-actin (#GTX26276, 1:10,000) and human IRS4 (#GTX61555, 1:1,000) from GeneTex.

Molecular mass (in KDa) is indicated next to each blot. Full scans of the Western blots are provided in Supplementary Fig. 11.

### Statistical analyses

Statistical tests are specified in the text and were calculated using the statistical programming language R, with the ‘Bioconductor' and ‘survival' packages. A *P*-value<0.05 was considered statistically significant.

### Data availability

All data sets generated or analysed during this study are included in this published article and its Supplementary Information files.

## Additional information

**How to cite this article:** Ikink, G. J. *et al*. IRS4 induces mammary tumorigenesis and confers resistance to HER2-targeted therapy through constitutive PI3K/AKT-pathway hyperactivation. *Nat. Commun.* **7,** 13567 doi: 10.1038/ncomms13567 (2016).

**Publisher's note**: Springer Nature remains neutral with regard to jurisdictional claims in published maps and institutional affiliations.

## Supplementary Material

Supplementary Information Supplementary Figures 1-11, Supplementary Methods and Supplementary References

Supplementary Data 1 Location and orientation of MMTV-proviral insertions from the insertional mutagenesis screens, also providing tumour ID, mouse strain background and genotype.

Supplementary Data 2 RSN-normalized log2-transformed IRS4 microarray expression values from 157 metastasized breast cancers and corresponding PAM50-based subtype, quantified by the microarray probe ILMN_1712774 of the Illumina HumanHT-12 v4 Expression BeadChip.
