# Supplementary material for: IRS4 induces mammary tumorigenesis and confers resistance to HER2-targeted therapy through constitutive PI3K/AKT-pathway hyperactivation
Source: Nat Commun. 2016 Nov 23;7:13567. doi: 10.1038/ncomms13567 (PMC5122961; doi:10.1038/ncomms13567)
Supplement: Supplementary Information — Supplementary Figures 1-11, Supplementary Methods and Supplementary References [file ncomms13567-s1.pdf]

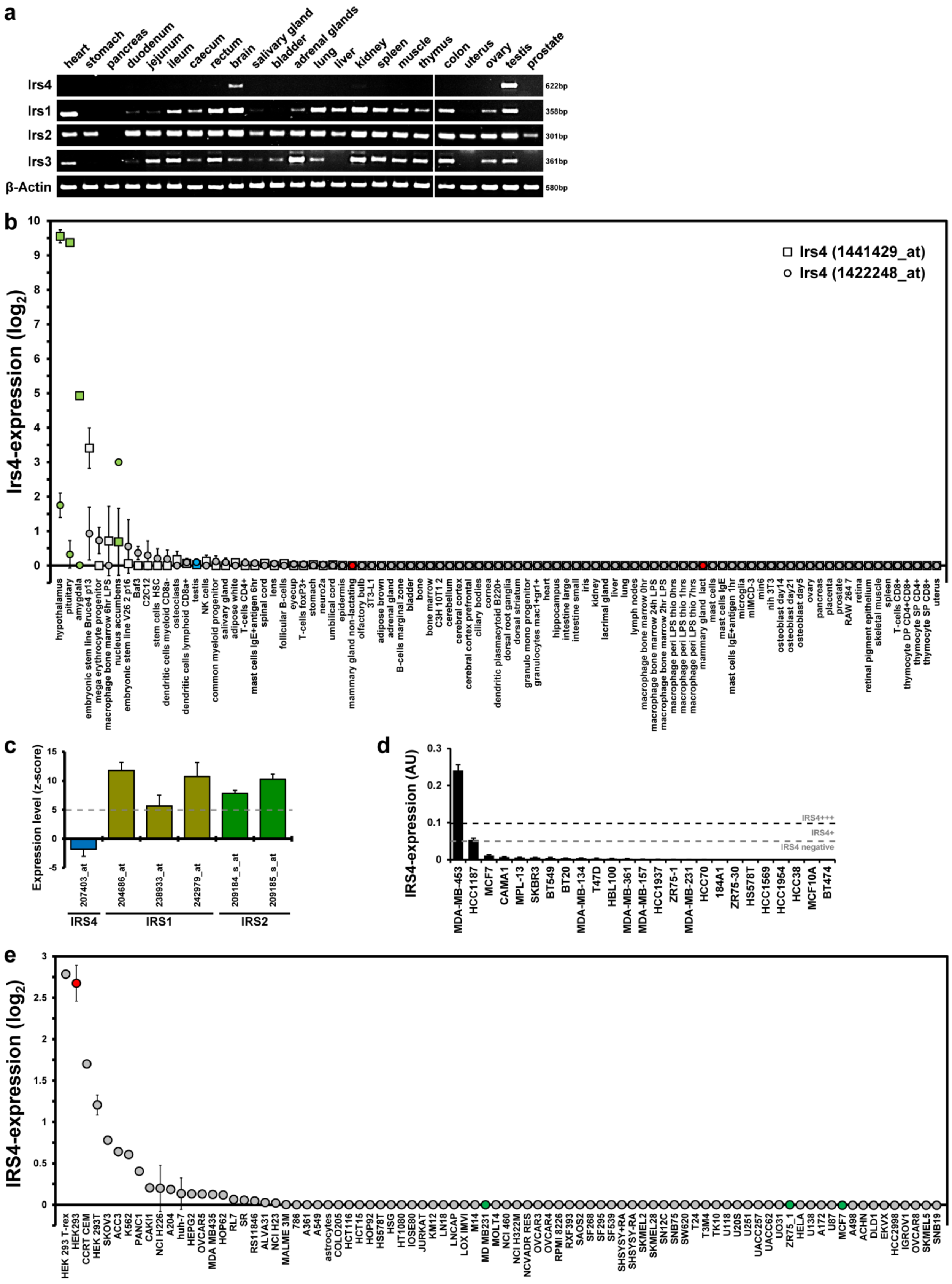
a
heart
stomach
pancreas
duodenum
jejunum
ileum
caecum
rectum
brain
salivary gland
bladder
adrenal glands
lung
liver
kidney
spleen
muscle
thymus
colon
uterus
ovary
testis
prostate
Irs4
Irs1
Irs2
Irs3
β-Actin
622bp
358bp
301bp
361bp
580bp
b
Irs4-expression (log2)
Irs4 (1441429_at)
Irs4 (1422248_at)
hypothalamus
pituitary
amygdala
embryonic stem line Bruce4 p13
mega erythrocyte progenitor
macrophage bone marrow 6hr LPS
nucleus accumbens
embryonic stem line V26 2 p16
Baf3
C2C12
stem cells HSC
dendritic cells myeloid CD8a-
osteoclasts
dendritic cells lymphoid CD8a+
testis
NK cells
common myeloid progenitor
salivary gland
adipose white
T-cells CD4+
mast cells IgE+antigen 6hr
spinal cord
lens
follicular B-cells
eyecup
T-cells foxP3+
stomach
neuro2a
umbilical cord
epidermis
mammary gland non-lactating
olfactory bulb
3T3-L1
adipose brown
adrenal gland
B-cells marginal zone
bladder
bone
bone marrow
C3H 10T1 2
cerebellum
cerebral cortex
cerebral cortex prefrontal
ciliary bodies
cornea
dendritic plasmacytoid B220+
dorsal root ganglia
dorsal striatum
granulo mono progenitor
granulocytes mac1+gr1+
heart
hippocampus
intestine large
intestine small
iris
kidney
lacrimal gland
liver
lung
lymph nodes
macrophage bone marrow 0hr
macrophage bone marrow 24h LPS
macrophage bone marrow 2hr LPS
macrophage peri LPS thio 0hrs
macrophage peri LPS thio 1hrs
macrophage peri LPS thio 7hrs
mammary gland lact
mast cells
mast cells IgE
mast cells IgE+antigen 1hr
microglia
mIMCD-3
min6
nih 3T3
osteoblast day14
osteoblast day21
osteoblast day5
ovary
pancreas
placenta
prostate
RAW 264 7
retina
retinal pigment epithelium
skeletal muscle
spleen
T-cells CD8+
thymocyte DP CD4+CD8+
thymocyte SP CD4+
thymocyte SP CD8+
uterus
c
Expression level (z-score)
207403_at
204686_at
238933_at
242979_at
209184_s_at
209185_s_at
IRS4
IRS1
IRS2
d
IRS4-expression (AU)
IRS4+++
IRS4+
IRS4 negative
MDA-MB-453
HCC1187
MCF7
CAMA1
MPL-13
SKBR3
BT549
BT20
MDA-MB-134
T47D
HBL100
MDA-MB-361
MDA-MB-157
HCC1937
ZR75-1
MDA-MB-231
HCC70
184A1
ZR75-30
HS578T
HCC1569
HCC1954
HCC38
MCF10A
BT474
e
IRS4-expression (log2)
HEK 293 T-rex
HEK293
CCRT CEM
HEK 293T
SKOV3
ACC3
K562
PANC1
CAKI1
NCI H226
A204
huh-7
HEPG2
OVCAR5
MDA MB435
HOP62
RL7
SR
RS11846
ALVA31
NCI H23
MALME 3M
786
A361
A549
astrocytes
COLO205
HCT116
HCT15
HOP92
HS578T
HSG
HT1080
IOSE80
JURKAT
KM12
LN18
LNCAP
LOX IMVI
M14
MD MB231
MOLT4
NCI 460
NCI H322M
NCVADR RES
OVCAR3
OVCAR4
RPMI 8226
RXF393
SAOS2
SF268
SF295
SF539
SHSYSY+RA
SHSYSY-RA
SKMEL2
SKMEL28
SN12C
SNB75
SW620
T24
T3M4
TK10
U118
U20S
U251
UACC257
UACC62
UO31
ZR75_1
HELA
U138
A172
U87
MCF7
A498
ACHN
DLD1
EKVX
HCC2998
IGROV1
OVCAR8
SKMEL5
SNB19

**Supplementary Figure 1. Expression pattern of *Irs4* in MMTV-induced tumours, murine and human tissues and cell lines.**

(**a**) mRNA-expression of *Irs*-gene family members in various tissues from wildtype BALB/c mice, determined by RT-PCR.

(**b**) *Irs4*-expression levels in 91 normal C57BL6 mouse tissues and cells, quantified by the indicated microarray probes of the Affymetrix Mouse Genome 430 2.0 Array, using the Mouse MOE430 Gene Atlas dataset[1] obtained from BioGPS[2]. Samples discussed in the results-section on **a** and Fig. 1e are highlighted: brain samples with high expression in green, testis in blue and mammary glands in red. Data represented as mean GCRMA-normalized $\log_2$-transformed signal values±s.d.

(**c**) Expression-levels of *IRS4*, *IRS1* and *IRS2* in human breast epithelium, quantified by the indicated microarray probes of the Affymetrix Human Genome U133 Plus 2.0 Array, using the Barcode on Normal Tissues dataset obtained from BioGPS[2]. Expression-levels shown as z-score[3], where >5 suggests expression in that tissue (indicated by grey dashed line). Data represented as mean±s.d.

(**d**) Expression of *IRS4*-mRNA in various human breast cancer cell lines, relative to *IRS4*-expression level in HEK-293 cells, determined by quantitative RT-PCR. Expression levels greater than median+s.d. (grey dashed line) were defined as positive (IRS4+) and greater than median+2*s.d. (black dashed line) as highly positive (IRS4+++).

(**e**) *IRS4*-expression levels in 84 human cell lines, quantified by microarray probe 207403_at of the Affymetrix Human Genome U133A Array, using the Human NCI60 Cell Lines dataset from BioGPS[2]. Cell lines tested in **d** are highlighted: green for breast cancer cells, red for HEK-293. Data represented as mean GCRMA-normalized $\log_2$-transformed signal values±s.d.

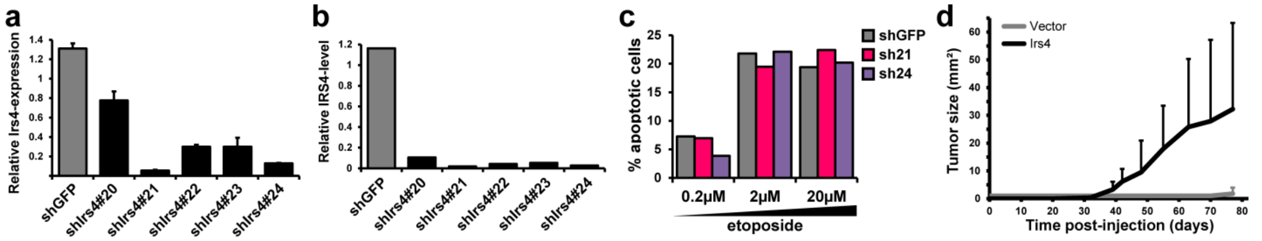


**Supplementary Figure 2. *Irs4*-expression in mammary cells drives tumour growth.**

(**a**) Knockdown of *Irs4*-expression in P3724-R4 tumour cells by the indicated short hairpin RNAs. *Irs4*-expression levels were determined by qRT-PCR and the results are presented relative to the expression-level in the parental cell line. The most effective two Irs4-shRNAs, shIrs4 #21 and #24, caused a 95% and 87% downregulation of *Irs4*-expression, respectively. Data are represented as mean+s.d. of triplicates.

(**b**) Quantification of IRS4-protein levels, corrected for loading and presented relative to the level in parental cells, derived from the Western blot shown in Fig. 2d. shIrs4 #21 and #24 reduced IRS4 to respectively 2% and 3% of the levels in the parental cells.

(**c**) Percentage of apoptotic cells (Annexin V-positive) in viable (propidium iodide-negative) populations of P3724-R4 cells as in Fig. 2g**,** cultured for 24 h in presence of indicated etoposide concentrations, determined by FACS.

(**d**) Tumour growth (mean+s.d.) in mice shown in Fig. 2i, subcutaneously injected in both flanks with $1\cdot10^6$ NMuMG-cells ectopically expressing *Irs4* (n = 5) and vector control cells (n = 5).

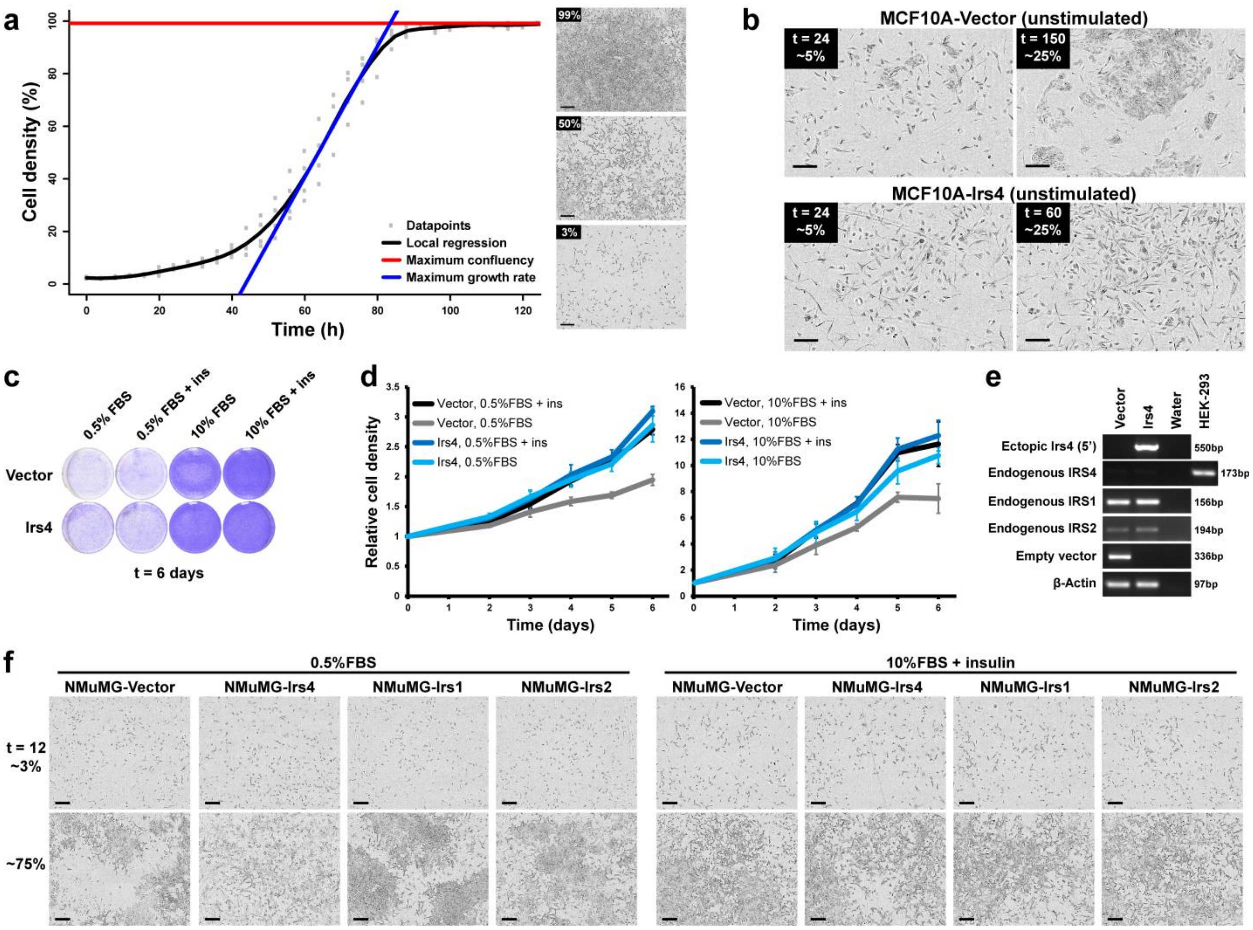


**Supplementary Figure 3. Effect of IRS4 on cell proliferation in mammary cells.**

(**a**) Example of local regression (black) of cell density over time of the depicted data points (grey). The maximum growth rate (blue) and maximum confluency (red) calculated from the local regression using the "cellGrowth" package in the programming language R are also shown. Representative graphical images obtained by the IncuCyte life cell imaging device and corresponding confluency are indicated on the right (scale bar = 200 µm).

(**b**) Representative images of unstimulated vector control and *Irs4*-transduced MCF10A-cells, obtained by the IncuCyte imager. Time point (t, in min) and corresponding confluency (%) is indicated (scale bar = 200 µm). Vector control cells display only sporadic growth, while MCF10A-Irs4 cells grow uniformly.

(**c**) Representative image of Crystal Violet stained NMuMG-cells, stably transfected with *Irs4* or empty vector, incubated for six days under the indicated growth conditions.

(**d**) Growth curves of the vector control and *Irs4*-transduced NMuMG-cells under the indicated conditions, derived from Crystal Violet stained cells as in **c**. Data are depicted as the mean±s.d. of four experiments using independently transduced cell pools.

(**e**) Expression of endogenous *IRS*-genes, ectopic *Irs4* and the empty pMSCV-vector construct in transduced MCF10A-cells, as determined by RT-PCR. *IRS1* and *IRS2*, but not *IRS4*, are endogenously expressed in these cells. HEK-293 cDNA was used as positive control for endogenous *IRS4*-expression.

(**f**) Representative images of indicated NMuMG-cells under the indicated conditions, obtained by the IncuCyte imager, with corresponding confluency (%) and time points (t, in min) indicated (scale bar = 200 µm). Time points at ~75% differ between cell lines (see Fig. 3d). NMuMG-Vector, NMuMG-Irs1 and NMuMG-Irs2 cells show only sporadic growth in 0.5%FBS, while MCF10A-Irs4 cells grow uniformly.

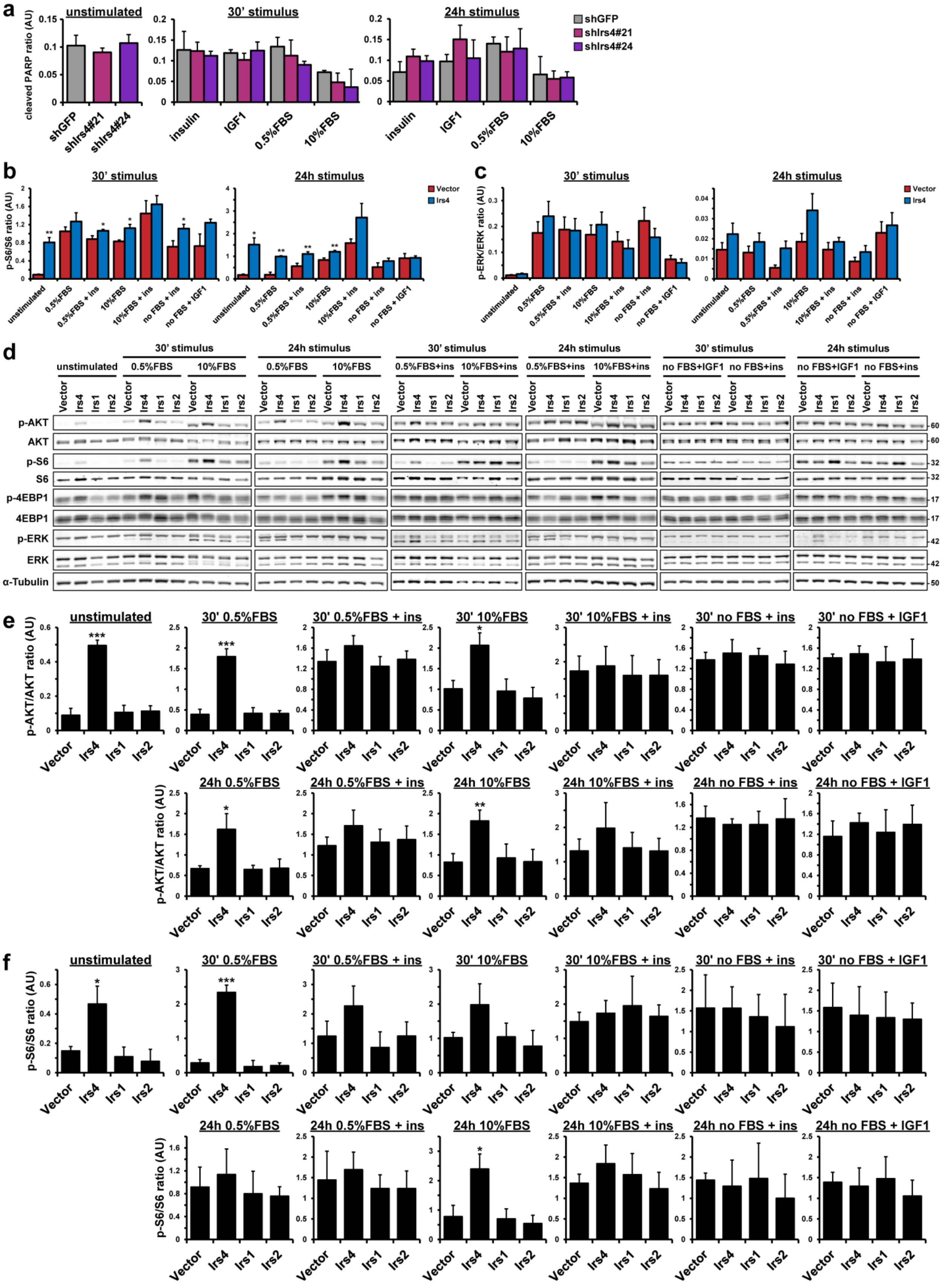

a
unstimulated
30' stimulus
24h stimulus
shGFP
shIrs4#21
shIrs4#24
cleaved PARP ratio (AU)
insulin
IGF1
0.5%FBS
10%FBS
b
c
Vector
Irs4
p-S6/S6 ratio (AU)
p-ERK/ERK ratio (AU)
unstimulated
0.5%FBS + ins
10%FBS + ins
no FBS + ins
no FBS + IGF1
d
p-AKT
AKT
p-S6
S6
p-4EBP1
4EBP1
p-ERK
ERK
α-Tubulin
Irs1
Irs2
0.5%FBS+ins
10%FBS+ins
no FBS+IGF1
no FBS+ins
e
p-AKT/AKT ratio (AU)
30' 0.5%FBS
30' 0.5%FBS + ins
30' 10%FBS
30' 10%FBS + ins
30' no FBS + ins
30' no FBS + IGF1
24h 0.5%FBS
24h 0.5%FBS + ins
24h 10%FBS
24h 10%FBS + ins
24h no FBS + ins
24h no FBS + IGF1
f

**Supplementary Figure 4. IRS4, not IRS1 and IRS2, constitutively stimulates PI3K/AKT-pathway signalling in mammary cells.**

(**a**) Ratios of cleaved PARP over full length PARP (mean+s.d.), quantified from three Western blots as shown in Fig. 4a. There are no significant differences between any of the ratios within each condition (Welch's t-test).

(**b**,**c**) Ratios of phosphorylated S6 (**b**) and ERK (**c**) over the respective total-proteins, calculated from Western blots of MCF10A-cells as shown in Fig. 4c. Data are represented as mean+s.d. and are based on three blots each loaded with lysates from independently transduced oligoclonal cell cultures. *p < 0.05, **p < 0.01 (Welch's t-test, compared to vector in each condition). There are no significant differences between any of the p-ERK/ERK-ratios.

(**d**) Representative Western blots showing phosphorylated (p-) and total-protein components of the PI3K/AKT and MAPK/ERK-pathways in NMuMG-cells transduced with *Irs4* or empty vector controls subjected to the indicated stimuli for 30 min or 24 h (ins = insulin).

(**e**,**f**) Ratios of phosphorylated over total AKT (**e**) and S6 (**f**) (mean+s.d.) from three blots as shown in **d**, loaded with lysates from independently transduced cell cultures. *p < 0.05, **p < 0.01, ***p < 0.001 (Welch's t-test, compared to vector in each condition).

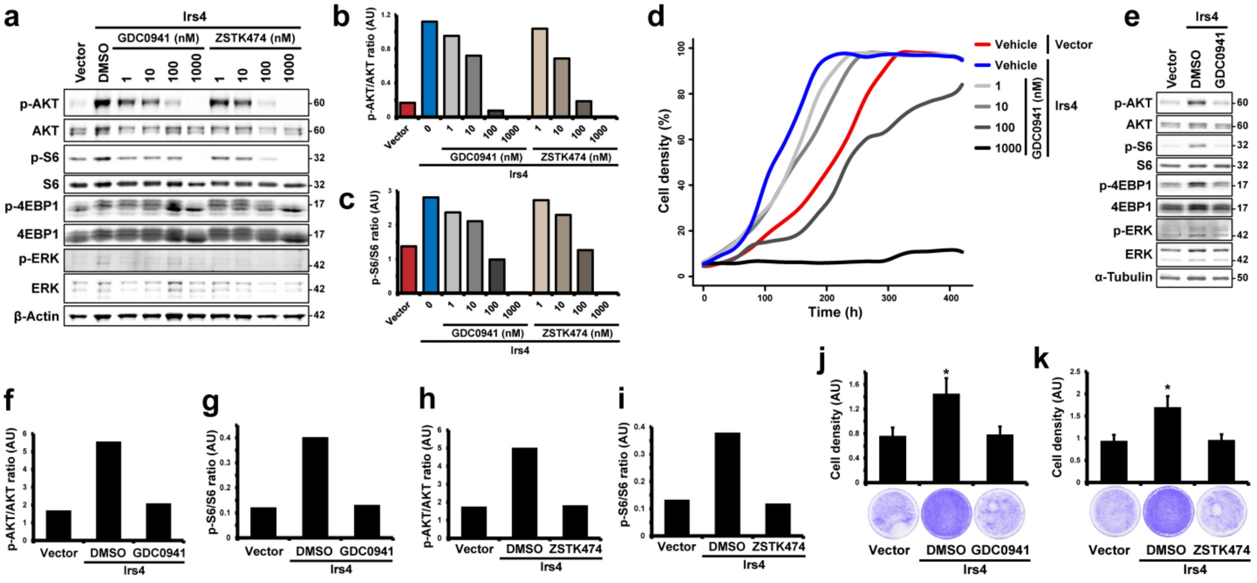


**Supplementary Figure 5. IRS4 induces constitutive stimulation of PI3K/AKT-pathway signalling increases cell proliferation in mammary epithelial cells.**

(**a**) Western blot showing phosphorylated (p-) and total-protein of components of the PI3K/AKT and MAPK/ERK-pathways from NMuMG-Irs4 cells grown in medium containing 0.5%FBS for 30 h in the presence of increasing concentrations of PI3K-specific inhibitors GDC0941 or ZSTK474, or in vehicle (DMSO). Lysates from NMuMG-cells transduced with empty vector were used as a control.

(**b**,**c**) Ratios of phosphorylated over total AKT (**b**) and S6 (**c**) from the blot in **a**.

(**d**) Growth curves of NMuMG-Irs4 cells in medium supplemented with 0.5%FBS and in the presence of increasing concentrations of the PI3K-specific inhibitor GDC0941, compared with vehicle treated and vector control cells. Growth was measured as cell density over time by the IncuCyte imager. Data are represented as local regression from quadruplicates.

(**e**) Representative Western blot of two independent experiments, showing phosphorylated and total-protein components of the PI3K/AKT and MAPK/ERK-pathways in NMuMG-Irs4 cells cultured 48 h in medium supplemented with 0.5%FBS and 200 nM GDC0941 or vehicle (DMSO). Cells transduced with empty vector were used as vector controls.

(**f**,**g**) Ratios of phosphorylated over total AKT (**f**) and S6 (**g**) from the Western blot in **e**.

(**h**,**i**) Ratios of phosphorylated over total AKT (**h**) and S6 (**i**) from the blot in Fig. 4f.

(**j**,**k**) Cell density of NMuMG-Irs4 cells cultured three days in medium supplemented with 0.5%FBS and 200 nM GDC0941 (**j**), 100 nM ZSTK474 (**k**) or vehicle (DMSO), and vehicle treated vector control cells. Data represented as mean+s.d. of three experiments, and are obtained from Crystal Violet cell staining assay. Representative images of the Crystal Violet staining are shown below the graphs.

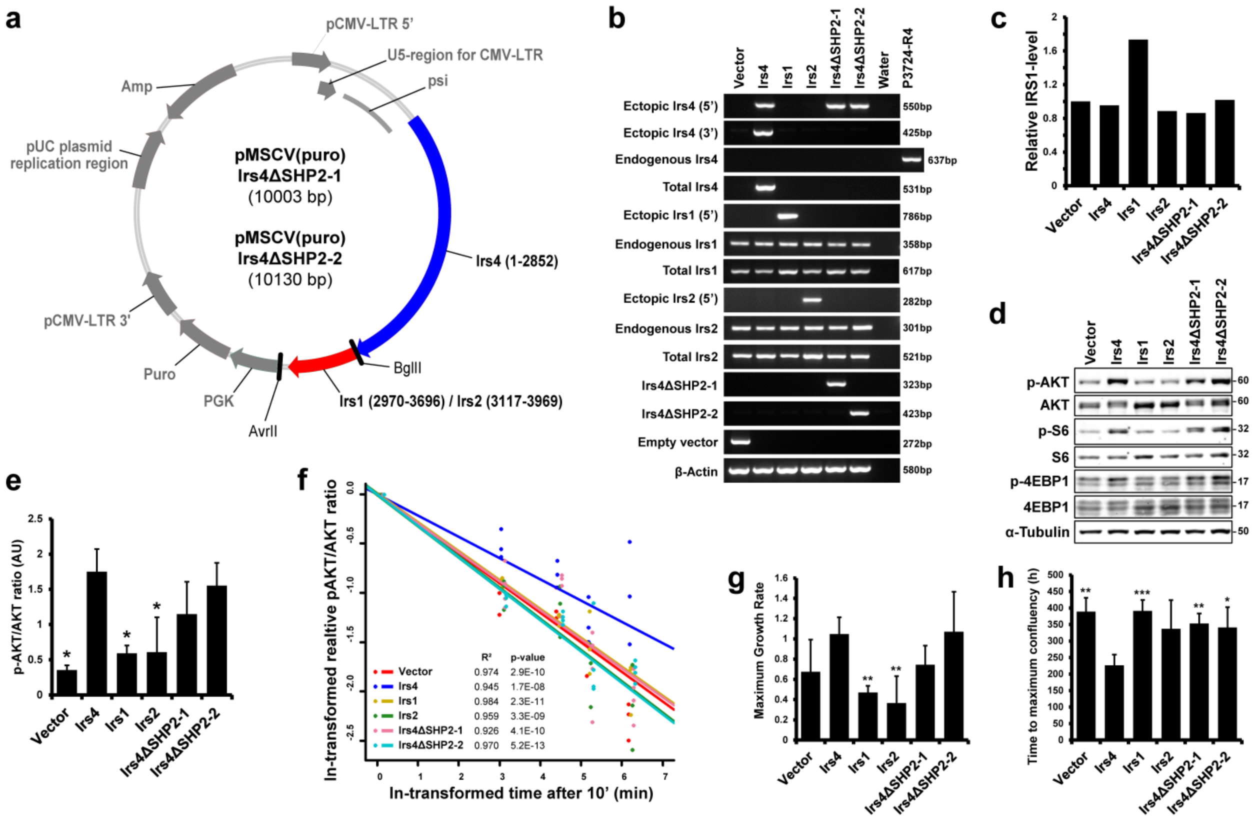


**Supplementary Figure 6. Lack of functional SHP2-binding domain permits constitutive signalling of IRS4.**

(**a**) Schematic map of the Irs4ΔSHP2-1 and Irs4ΔSHP2-2 constructs. The 5' translated region of *Irs4* is indicated in blue and the SHP2-domain from the 3'-regions of *Irs1* or *Irs2* in red. Following the names of the inserted cDNAs, the nucleotide positions of the inserts are provided, based on the coding sequences of each cDNA (i.e. counting from the first A of ATG in the translational start site). Restriction sites of BglII and AvrII that were used for cloning are also shown.

(**b**) mRNA-expression of *Irs*-family members, *Irs4*-recombinants and empty vector construct transduced in NMuMG-cells used in the experiments as determined by RT-PCR. For assessment of ectopic expression, the primer annealing to the vector backbone was located either upstream (5') or downstream (3') of the coding sequence of the insert. For specifically

analysing the expression of the endogenous products, one of the primers was located in the untranslated region (UTR) of the genes, while the other was located in the translated region. Where assessing total *Irs*-gene expression (ectopic and endogenous), we used primers pairs located in the translated region of the genes. P3724-R4 tumour cell line cDNA was used as positive control for endogenous *Irs4*-expression.

(**c**) Quantification of IRS1-protein levels in the Western blot shown in Fig. 5b, corrected for loading and presented relative to the level in vector control cells. Although IRS1 is endogenously expressed in these cells, additional ectopic expression of the gene increases IRS1-levels with ~70%.

(**d**) Representative Western blots of three experiments, showing phosphorylated (p-) and total-protein of components in the PI3K/AKT-pathway of the same transduced NMuMG-cells as in Fig. 5b, after stimulation with 0.5% FBS for 10 min.

(**e**) Ratios phosphorylated AKT over total AKT, calculated from Western blots as those shown in **d**. Data are represented as mean+s.d. from three blots. The gels were loaded with lysates from independently transduced cells. *$p < 0.05$ (Welch's t-test, compared to Irs4).

(**f**) Linear regression of ln-transformed phosphorylated AKT over total AKT ratios vs time after stimulation, relative to peak signalling at 10 min stimulation, determined from three independent blots of Fig. 5f. The transformed data points are fitted in a linear model and associated $R^2$-values and model p-values are indicated. The 24 h time point was omitted for the linear model fit, as relative pAKT/AKT ratios increased again at this time point. For the same reason, the 8 h time point was additionally omitted to fit the linear model for NMuMG-Irs4, but was nonetheless plotted in the figure.

(**g**,**h**) Quantification of maximum growth rates (**g**) and times until maximum confluency was reached (**h**) of NMuMG-cells transduced with the indicated constructs. The data were derived from growth curves represented in Fig. 5h and were calculated as outlined in Supplementary

Fig. 3a. Data represented as mean+s.d. of quadruplicates. *$p < 0.05$, **$p < 0.01$, ***$p < 0.001$ (Welch's t-test, compared to Irs4).

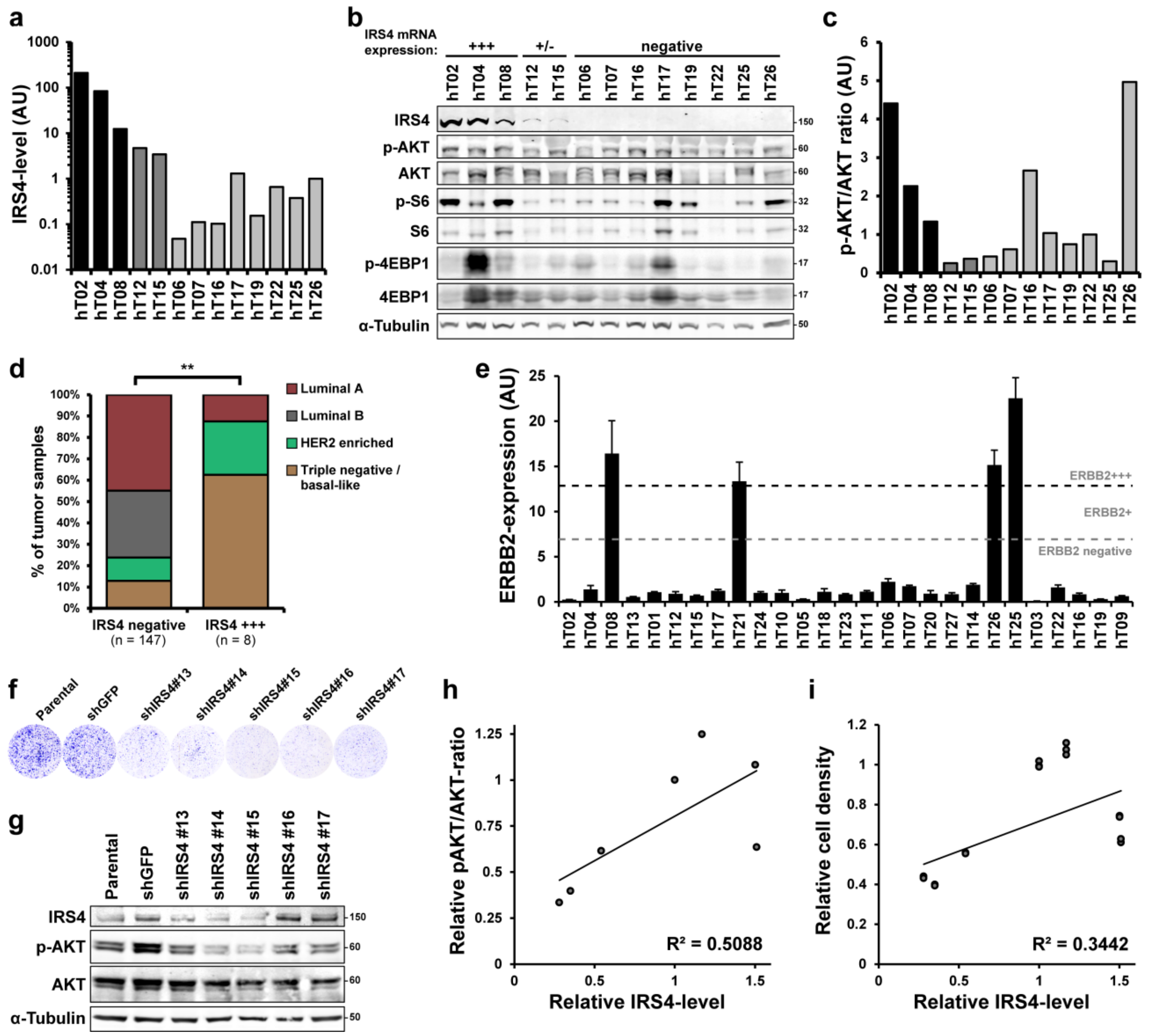


**Supplementary Figure 7. IRS4 in human breast cancer patient material and cells.**

(**a**) Quantification of IRS4-protein levels (log-scale), corrected for loading, derived from the Western blot shown in Fig. 6b.

(**b**) Western blot analysis showing phosphorylated (p-) and total protein levels of major players in the PI3K/AKT-pathway from the primary human breast tumours shown in Fig. 6b.

(**c**) Ratios of phosphorylated over total AKT, calculated from the blot in **b**, after normalization to the loading control levels (α-tubulin).

(**d**) Distribution of the IRS4-negative vs IRS4+++ tumours using the threshold depicted in Fig. 6c over the indicated PAM50-based clinical subtypes. $^{**}p < 0.01$ (Pearson's Chi-squared test).

(**e**) Expression of *ERBB2*-mRNA in the 27 random human primary breast carcinomas from Fig. 6a, determined by qRT-PCR. Expression levels greater than median+s.d. (grey dashed line) were determined as positive (ERBB2+) and greater than median+2*s.d. (black dashed line) as highly positive (ERBB2+++).

(**f**) Representative images of triplicates, showing Crystal Violet staining of MDA-MB-453 cells, transduced with the indicated shRNAs, allowed to grow for 10 days.

(**g**) Western blot showing IRS4-protein, and phosphorylated (p-) and total-AKT from HCC1187-cells transduced with the indicated shRNAs.

(**h**) Quantified IRS4-levels plotted against ratios of phosphorylated AKT over total AKT, derived from the blot in **g**. Linear regression and correlated $R^2$-values show a positive correlation.

(**i**) Quantified IRS4-levels, derived from the blot in **g**, plotted against their cell proliferation rate, determined by the Crystal Violet staining of the same cells allowed to grow for 6 days (in triplicate). Linear regression and correlated $R^2$-values show a positive correlation.

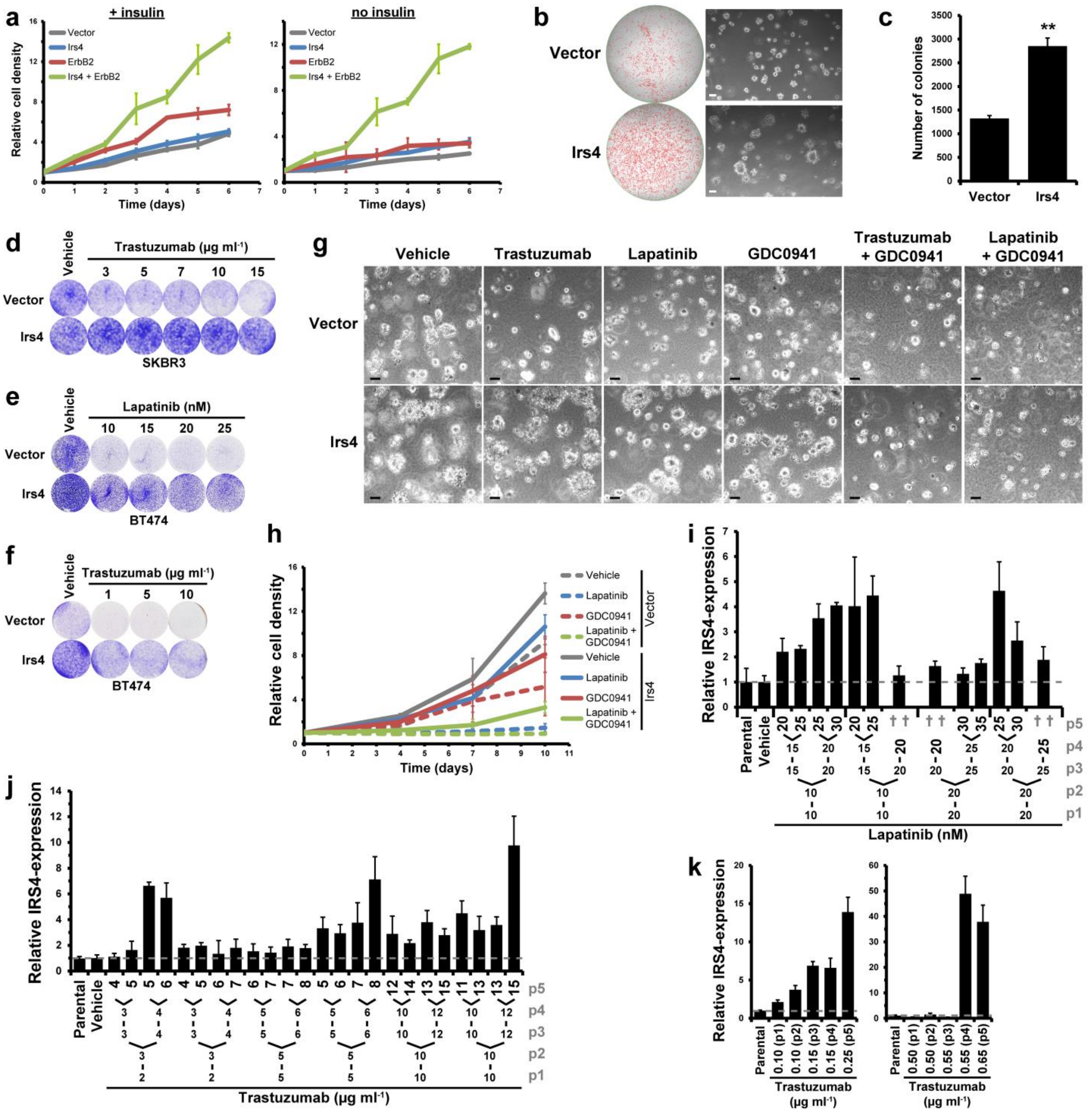


**Supplementary Figure 8. IRS4 synergizes with ERBB2 (HER2) and induces resistance to Trastuzumab and Lapatinib.**

(**a**) Growth curves of NMuMG-cells, stably transfected with *Irs4* and/or *ErbB2*, or empty vector, derived from Crystal Violet staining. Cells were cultured in medium supplemented with

0.5%FBS, and with (left) or without (right) insulin. Data depicted are the mean±s.d. of three experiments using independently transduced cell pools.

(**b**) Representative whole well scans and phase-contrast micrographs of three soft agar growth experiments of BT474-cells, stably transfected with *Irs4* or empty vector. The cells were allowed to grow in soft agar for two weeks. (Scale bar = 50 µm).

(**c**) Quantification of anchorage-independent growth by the GelCount colony counter of three independently transduced BT474-cell cultures as shown in **b**. Data are represented as mean+s.d.. **$p < 0.01$ (Welch's t-test, compared to vector).

(**d**,**e**,**f**) Representative images of four independent experiments, showing Crystal Violet staining of SKBR3-cells (**d**) and BT474-cells (**e**,**f**), stably transfected with *Irs4* or empty vector, allowed to grow for 11 days in presence of the indicated Lapatinib (**e**) or Trastuzumab (**d**,**f**) concentrations, or vehicle.

(**g**) Representative phase-contrast micrographs of anchorage-independent growth of BT474-cells from the soft agar assay depicted in Fig. 7f (scale bar = 50 µm).

(**h**) Growth curves of SKBR3-cells, stably transfected with *Irs4* or empty vector, treated with 50 nM Lapatinib and/or 50 nM GDC0941, or vehicle (DMSO), derived from Crystal Violet staining. Data depicted are the mean±s.d. of three experiments using independently transduced cell pools.

(**i**,**j**) *IRS4*-expression levels of SKBR3-cells (lanes marked 'Parental') and subcultures of the same cell lines cultured for five passages in presence of increasing concentrations of Lapatinib (**i**) or Trastuzumab (**j**), relative to vehicle-treated cells (indicated by grey dashed lines). *IRS4*-expression levels were determined by qRT-PCR and are represented as mean+s.d. of triplicates. Passage number and corresponding drug concentrations are indicated. The †-symbol indicates that the cells did not survive the increase in concentration in the fifth passage, therefore *IRS4*-expression of the fourth passage was measured instead. The cells were initially also cultured in the presence of 40 nM Lapatinib in passage 1, but the BT474-cells did not

survive this concentration even for a single passage, while SKBR3-cells did not survive more than two passages. Hence, these data are not shown.

(**k**) Relative *IRS4*-expression levels from cultures in Fig. 7i, showing all five passages of a culture acquiring *IRS4*-expression early (left) and late (right).

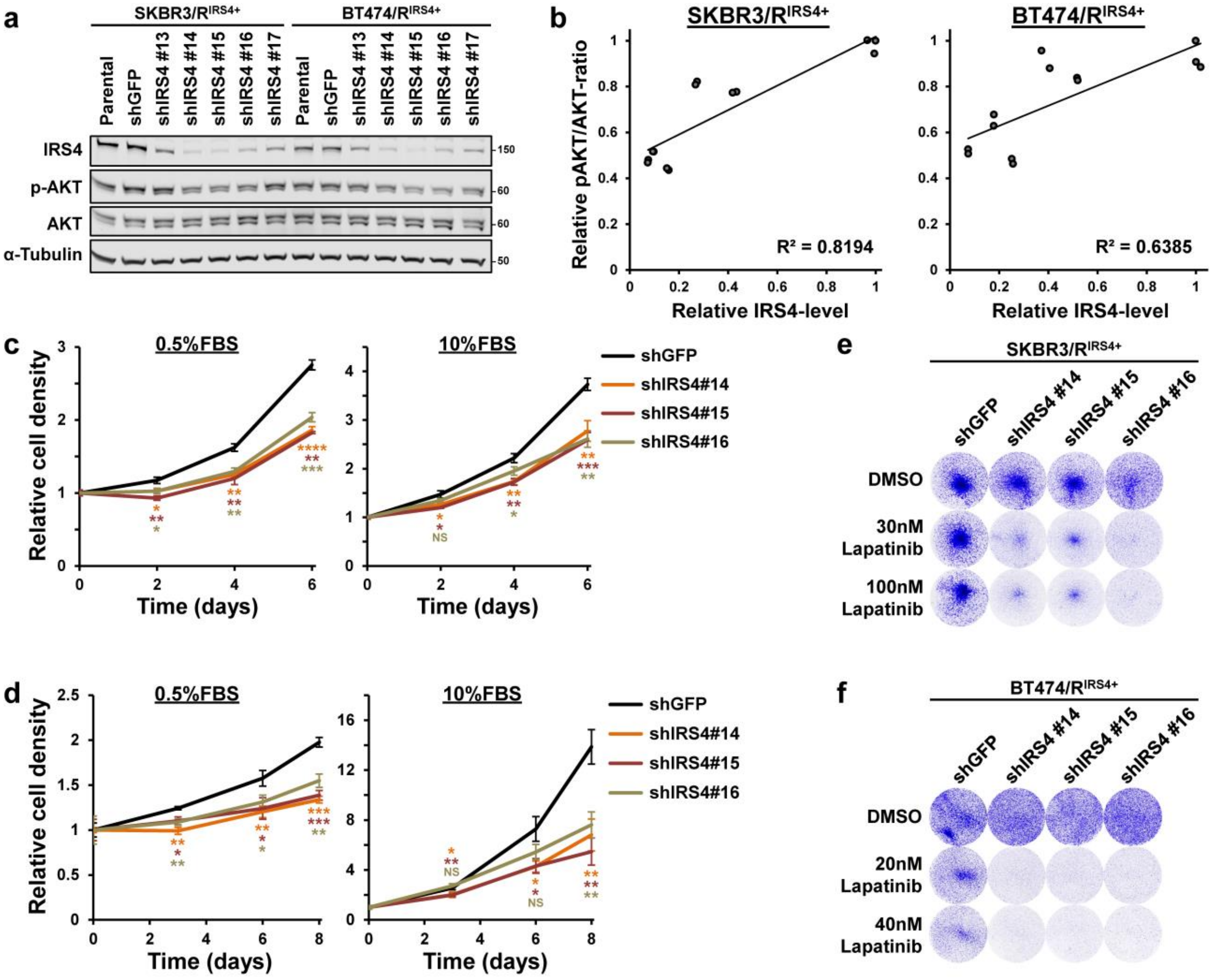


**Supplementary Figure 9. Knockdown of endogenous *IRS4* reduces PI3K/AKT-pathway activation, cell proliferation and Lapatinib resistance.**

(**a**) Western blots of IRS4 and phosphorylated (p-) and total-AKT from SKBR3/R$^{IRS4+}$ and BT474/R$^{IRS4+}$ cells transduced with the indicated shRNAs.

(**b**) Quantified IRS4-levels plotted against ratios of phosphorylated AKT over total AKT, derived from the blot in **a** (and duplicate). Linear regression and correlated $R^2$-values show a positive correlation.

(**c**,**d**) Growth curves of SKBR3/R$^{IRS4+}$ (**c**) and BT474/R$^{IRS4+}$ cells (**d**), transduced with the indicated shRNAs giving the strongest knockdown of IRS4, compared to shGFP-controls,

cultured in 0.5% or 10% serum (FBS). Data depicted as the mean±s.d. of three Crystal Violet staining experiments. NS, not significant; *$p < 0.05$, **$p < 0.01$, ***$p < 0.001$ (Welch's t-test, compared to shGFP).

(**e**,**f**) Representative images of three experiments, showing Crystal Violet staining of SKBR3/$R^{IRS4+}$ (**e**) and BT474/$R^{IRS4+}$ cells (**f**) as in **c** and **d**, allowed to grow for 6 days in presence of the indicated Lapatinib concentrations, or vehicle (DMSO).

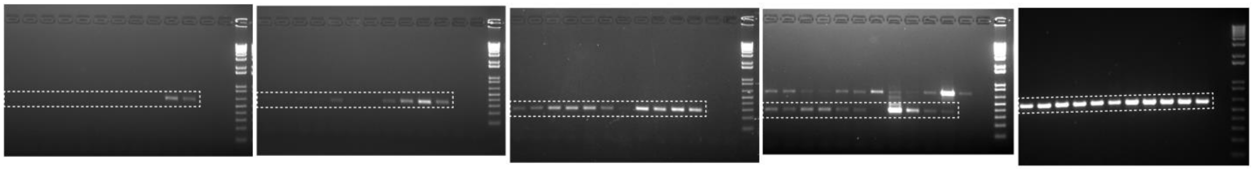

**Supplementary Figure 10. Full size gels corresponding to Fig. 1e**

See the legend of Fig. 1e for details.

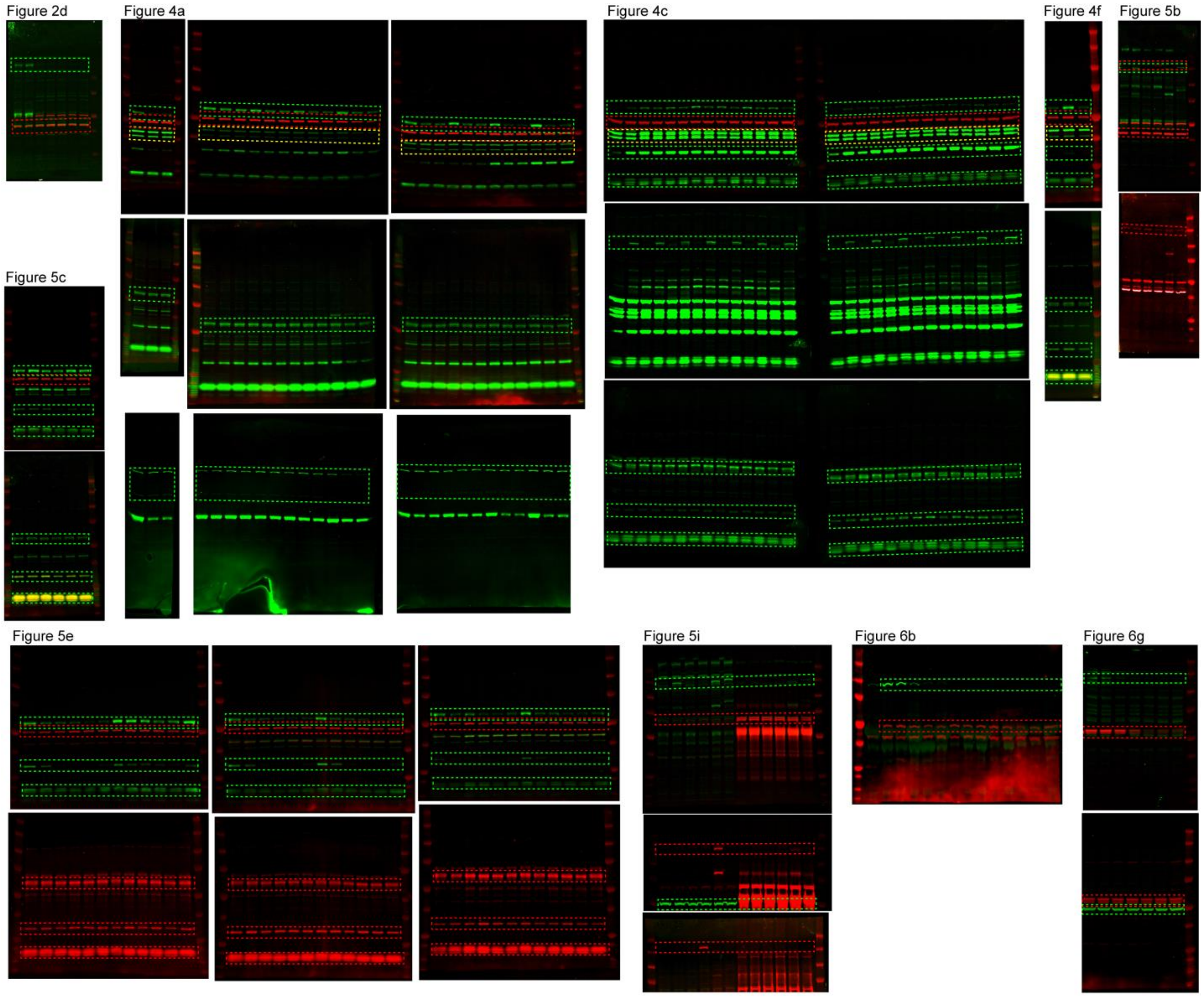


**Supplementary Figure 11. Full sized scans of the Western blots corresponding to Fig. 2d, 4a, 4c, 4f, 5b, 5c, 5e, 5i, 6b and 6g.**

See the legends of the associated Figures for details.

## SUPPLEMENTARY METHODS

### Supplementary Cell Lines and Culture Conditions

184A1, BT549, CAMA1, HBL100, HCC1569, HCC1937, HCC1954, HCC38, HCC70, HEK293, HS578T, MCF7, MDA-MB-361, T47D and ZR75-30 were purchased from ATCC and the ZR75-1 cell line from Centocor. BT20, MDA-MB-134 and MDA-MB-157 were a kind gift from Dr. J. Taylor-Pappadimitriou (Guy's Hospital, London) and the MDA-MB-231 cell line was kindly provided by Dr. M.M.K. Mareel (University of Ghent). The MPL-13 cell line is an in-house established cell line from pleural effusion of a breast cancer patient. 184A1 were cultured in DMEM/F-12 medium supplemented with 10% FBS, 50 units $ml^{-1}$ penicillin and 50 µg $ml^{-1}$ streptomycin (PenStrep). HCC1569, HCC1937, HCC1954, HCC38, HCC70 and ZR75-30 cells were cultured in RPMI 1640 medium (Gibco) supplemented with 10% FBS and PenStrep. All other cells were cultured in DMEM supplemented with 10% FBS and PenStrep. SKBR3-medium was additionally supplemented with 5 µg $ml^{-1}$ insulin. All cell lines were never cultured for more than eight passages upon receipt and were routinely tested for Mycoplasma (Hoechst staining and PCR).

### Supplementary Apoptosis Assays

$0.8 \cdot 10^6$ P3724-R4 cells were allowed to attach overnight to a T25 flask (BD Falcon) in DMEM/F-12 medium supplemented with 10% FBS, PenStrep, 5 µg $ml^{-1}$ insulin, 200 ng $ml^{-1}$ hydrocortisone and 20 ng $ml^{-1}$ EGF, after which 0.2 µM, 2 µM or 20 µM etoposide was added to the medium. After 24 h, cell exposed to 2 µM or 20 µM etoposide showed clear blebbing, upon which the cells were released by EDTA-free trypsin-250 solution and stained with Annexin V and PI using the Annexin V-FITC Apoptosis Detection Kit (Abcam), following manufacturers' instructions, and analysed using a Beckton Dickinson LSRII FACS analyser.

**Supplementary Cell Proliferation Assays**

25,000 or 80,000 cells per well were seeded in 12-wells plates (Corning) for 6-8 day or 3-day experiments, respectively. Cells were allowed to attach for 6 h, washed with DMEM/F-12 medium only supplemented with 50 units $ml^{-1}$ penicillin and 50 µg $ml^{-1}$ streptomycin (Gibco) and then grown under the indicated conditions. At the indicated time points, the cells were fixed with 4% Formaldehyde solution (Klinipath). Fixed cells of all time points were stained simultaneously with 0.1% Crystal Violet solution (Sigma-Aldrich) and imaged using a desktop scanner (Epson). Cell proliferation was quantified by extracting the dye with 10% acetic acid and measuring the absorbance at 590 nm using the Infinite plate reader (TECAN).

## SUPPLEMENTARY REFERENCES


1. Lattin JE*, et al.* Expression analysis of G Protein-Coupled Receptors in mouse macrophages. *Immunome research* **4**, 5 (2008).

2. Wu C*, et al.* BioGPS: an extensible and customizable portal for querying and organizing gene annotation resources. *Genome biology* **10**, R130 (2009).

3. McCall MN, Uppal K, Jaffee HA, Zilliox MJ, Irizarry RA. The Gene Expression Barcode: leveraging public data repositories to begin cataloging the human and murine transcriptomes. *Nucleic Acids Research* **39**, D1011-D1015 (2011).
